# Associations Between Systolic Interarm Differences in Blood Pressure and Cardiovascular Disease Outcomes and Mortality: Individual Participant Data Meta-Analysis, Development and Validation of a Prognostic Algorithm: The INTERPRESS-IPD Collaboration

**DOI:** 10.1161/HYPERTENSIONAHA.120.15997

**Published:** 2020-12-21

**Authors:** Christopher E. Clark, Fiona C. Warren, Kate Boddy, Sinead T.J. McDonagh, Sarah F. Moore, John Goddard, Nigel Reed, Malcolm Turner, Maria Teresa Alzamora, Rafel Ramos Blanes, Shao-Yuan Chuang, Michael Criqui, Marie Dahl, Gunnar Engström, Raimund Erbel, Mark Espeland, Luigi Ferrucci, Maëlenn Guerchet, Andrew Hattersley, Carlos Lahoz, Robyn L. McClelland, Mary M. McDermott, Jackie Price, Henri E. Stoffers, Ji-Guang Wang, Jan Westerink, James White, Lyne Cloutier, Rod S. Taylor, Angela C. Shore, Richard J. McManus, Victor Aboyans, John L. Campbell

**Affiliations:** 1 From the Primary Care Research Group, Institute of Health Services Research (C.E.C., F.C.W., S.T.J.M., S.F.M., R.S.T., J.L.C.), University of Exeter Medical School, College of Medicine & Health, Devon, England; 2 Patient and Public Involvement Team, PenCLAHRC (K.B., J.G., N.R., M.T.), University of Exeter Medical School, College of Medicine & Health, Devon, England; 3 Institute of Biomedical and Clinical Science (A.H.), University of Exeter Medical School, College of Medicine & Health, Devon, England; 4 Unitat de Suport a la Recerca Metropolitana Nord, Fundació Institut Universitari per a la recerca a l’Atenció Primària de Salut Jordi Gol i Gurina (IDIAPJGol), Mataró, Spain (M.T.A.); 5 Unitat de Suport a la Recerca Girona, Fundació Institut Universitari per a la recerca a l’Atenció Primària de Salut Jordi Gol i Gurina (IDIAPJGol), Institut d’Investigació Biomèdica de Girona (IdIBGi), Department of Medical Sciences, School of Medicine, University of Girona, Spain (R.R.B.); 6 Institute of Population Health Sciences, National Health Research Institutes (NHRI), Taiwan, R.O.C (S.-Y.C.); 7 Department of Family Medicine and Public Health, University of California, San Diego, School of Medicine, La Jolla (M.C.); 8 Vascular Research Unit, Department of Vascular Surgery, Viborg Regional Hospital, Heibergs Allé 4, 8800 Viborg, Denmark (M.D.); 9 Department of Clinical Medicine, Aarhus University, Denmark (M.D.); 10 Department of Clinical Science in Malmö, Lund University, Sweden (G.E.); 11 Institute of Medical Informatics, Biometry and Epidemiology, University Hospital Essen, Germany (R.E.); 12 Wake Forest School of Medicine, NC (M.E.); 13 National Institute on Aging, Baltimore MD (L.F.); 14 INSERM U1094 & IRD, Tropical Neuroepidemiology, Institut d’Epidémiologie et de Neurologie Tropicale (IENT), Faculté de Médecine de l’Université de Limoges, Limoges Cedex, France (M.G., V.A.); 15 Lípid and Vascular Risk Unit, Internal Medicine Service, Carlos III, La Paz Hospital, Madrid, Spain (C.L.); 16 Department of Biostatistics, University of Washington, WA (R.L.M.); 17 Northwestern University Feinberg School of Medicine, Chicago, IL (M.M.M.); 18 Usher Institute of Population Health Sciences and Informatics, University of Edinburgh, Scotland (J.P.); 19 Department of Family Medicine, CAPHRI Care and Public Health Research Institute, Maastricht University, the Netherlands (H.E.S.); 20 Centre for Epidemiological Studies and Clinical Trials, Shanghai Key Laboratory of Hypertension, The Shanghai Institute of Hypertension, Ruijin Hospital, Shanghai Jiaotong University School of Medicine, China (J.-G.W.); 21 Department of Vascular Medicine, University Medical Center Utrecht, the Netherlands (J. Westerink); 22 DECIPHer, Centre for Trials Research, College of Biomedical and Life Sciences, Cardiff University, Wales (J. White); 23 Département des Sciences Infirmières, Université du Québec à Trois-Rivières, Canada (L.C.); 24 MRC/CSO Social and Public Health Sciences Unit & Robertson Centre for Biostatistics, Institute of Health and Well Being, University of Glasgow, Scotland (R.S.T.); 25 NIHR Exeter Clinical Research Facility, Royal Devon and Exeter Hospital and University of Exeter College of Medicine & Health, England (A.C.S.); 26 Nuffield Department of Primary Care Health Sciences, University of Oxford, England (R.J.M.); 27 Department of Cardiology, Dupuytren University Hospital, and Inserm 1094, Tropical Neuroepidemiology, Limoges, France (V.A.).

**Keywords:** blood pressure, cardiovascular disease, meta-analysis, mortality, risk

## Abstract

Systolic interarm differences in blood pressure have been associated with all-cause mortality and cardiovascular disease. We undertook individual participant data meta-analyses to (1) quantify independent associations of systolic interarm difference with mortality and cardiovascular events; (2) develop and validate prognostic models incorporating interarm difference, and (3) determine whether interarm difference remains associated with risk after adjustment for common cardiovascular risk scores. We searched for studies recording bilateral blood pressure and outcomes, established agreements with collaborating authors, and created a single international dataset: the Inter-arm Blood Pressure Difference - Individual Participant Data (INTERPRESS-IPD) Collaboration. Data were merged from 24 studies (53 827 participants). Systolic interarm difference was associated with all-cause and cardiovascular mortality: continuous hazard ratios 1.05 (95% CI, 1.02–1.08) and 1.06 (95% CI, 1.02–1.11), respectively, per 5 mm Hg systolic interarm difference. Hazard ratios for all-cause mortality increased with interarm difference magnitude from a ≥5 mm Hg threshold (hazard ratio, 1.07 [95% CI, 1.01–1.14]). Systolic interarm differences per 5 mm Hg were associated with cardiovascular events in people without preexisting disease, after adjustment for Atherosclerotic Cardiovascular Disease (hazard ratio, 1.04 [95% CI, 1.00–1.08]), Framingham (hazard ratio, 1.04 [95% CI, 1.01–1.08]), or QRISK cardiovascular disease risk algorithm version 2 (QRISK2) (hazard ratio, 1.12 [95% CI, 1.06–1.18]) cardiovascular risk scores. Our findings confirm that systolic interarm difference is associated with increased all-cause mortality, cardiovascular mortality, and cardiovascular events. Blood pressure should be measured in both arms during cardiovascular assessment. A systolic interarm difference of 10 mm Hg is proposed as the upper limit of normal.

**Registration:**

URL: https://www.crd.york.ac.uk/prospero/display_record.php?ID=CRD42015031227

## Abstract 2

Supplemental Digital Content is available in the text.

Cardiovascular disease is the leading cause of death worldwide, and hypertension is a major risk factor.^1^ Benefits of hypertension treatment are greatest for individuals with the highest estimated cardiovascular risk,^2^ but the majority of events occur in individuals with low to medium cardiovascular risk scores, therefore, novel additional risk markers may help to identify individuals most likely to benefit from preventative measures.^3^ Cardiovascular risk assessments generally take place in primary care settings,^4^ so ideally risk markers should be easily measured, with low acquisition costs for practical implementation.^5^

Measurement of blood pressure (BP) in both arms is easily achieved. International hypertension guidelines recommend checking BP in both arms and subsequently monitoring BP on the higher reading arm.^6–9^ Guidelines also acknowledge the association of systolic interarm BP differences (IAD) with cardiovascular risk.^6,7^ Systolic IADs ≥10 mm Hg arise in 11% of hypertensive people and in 4% of the general population.^10^ Cross-sectional studies report higher prevalence of systolic IAD in the presence of diabetes and cardiovascular, cerebrovascular, or peripheral arterial diseases.^10–12^ IAD, after adjustment for BP, is associated with increased left ventricular mass,^13^ greater arterial stiffness,^13–15^ diabetic nephropathy,^16,17^ and retinopathy.^16^ Study-level meta-analyses have observed associations of IAD with cardiovascular and all-cause mortality and with increased cardiovascular event rates.^12,18^ Independent risk has also been demonstrated for IAD after adjustment for Framingham risk scores.^19–21^

However, study-level meta-analyses are limited in their ability to draw conclusions since they combine studies with different patient characteristics, methodological choices, and analytical approaches. Such limitations can be minimized by using individual participant data (IPD).

We established the Inter-arm Blood Pressure Difference - Individual Participant Data (INTERPRESS-IPD) Collaboration to (1) to conduct the first international IPD meta-analysis to examine the independent association of systolic IAD with risk for cardiovascular events, all-cause mortality, and cardiovascular mortality; (2) develop and validate new cardiovascular risk prediction models that incorporate IAD; and (3) determine whether IAD predicts additional risk beyond existing cardiovascular risk scores.

## Methods

Data-sharing agreements were signed with each collaborating study lead author, and an Independent Monitoring Group oversaw conduct. Under terms of the data-sharing agreements the INTERPRESS-IPD dataset is not freely available but can be discussed with the corresponding author on reasonable request. The study protocol is registered with the international prospective register of systematic reviews (PROSPERO) and has been published.^22^ This study was conducted in accordance with the PRISMA-IPD (Preferred Reporting Items for Systematic Reviews and Meta-Analyses of IPD) statement.^23^

### Literature Search and Study Identification

Medline, Old Medline, Medline in process, Embase, and CINAHL were searched (Table S1 in the Data Supplement) for prospective studies reporting BP measurement in both arms. Unpublished data were sought from collaborators and the OpenGrey database. Searches were undertaken on April 19, 2016 with monthly updates until January 2017; dataset submission closed on April 7, 2017. Citations were screened in Covidence (Veritas Health Innovation, Melbourne, Australia) by 2 coauthors (S.F. Moore and S.T.J. McDonagh, L. Cloutier, C.E. Clark, J.L. Campbell, R.J. McManus, or A.C. Shore) independently; conflicts were resolved by discussion, with adjudication by C.E. Clark if unresolved.

Observational longitudinal studies or randomized controlled trials without BP lowering interventions, recording recruitment BP in both arms, were eligible. Inclusion criteria were participants aged ≥18 years, recruited from community, primary care, or general clinic settings. Primary outcomes were all-cause mortality, cardiovascular mortality, or fatal and nonfatal cardiovascular events. Selective cohorts such as vascular clinics were excluded. Participant and study-level characteristics known to relate to cardiovascular risk or BP and IAD measurement were sought. These included age, sex, ethnic group, body mass index, smoking status, total and HDL (high-density lipoprotein) cholesterol, and preexisting diagnoses of hypertension, diabetes, cardiovascular, renal and peripheral arterial diseases, cerebrovascular disease, and methods of BP measurement.

### Data Extraction and Preparation

Eligible study authors were invited to share data; nonresponders received 2 reminders. Anonymized data were securely transferred to the University of Exeter server and cleaned with reference to published data and author discussion, where necessary. Datasets were combined using Stata v14.0 (StataCorp, TX). For randomized controlled trials, we excluded interaction effects on outcomes between systolic IAD and treatment before including both trial arms in the IPD dataset. To overcome varying classifications of ethnicity, we mapped participants to the most commonly observed ethnic groups, seeking author and expert advice where required (Table S2). Two authors (S.T.J. McDonagh, L. Cloutier, or C.E. Clark) independently assessed study quality using a modified Quality Improvement in Prognostic Studies tool (Table S3).^24^

### Statistical Analysis

We defined IAD as absolute difference between the first pair of systolic arm BP readings. Where baseline BP was also measured separately to paired measurements during recruitment, we used this measurement in adjusted models. If only paired readings existed we adopted the higher reading arm BP.^6,7^ For the inferential analyses and modeling, follow-up analysis times were truncated to 10 years. Cardiovascular events were defined as first occurrence of myocardial infarction, physician confirmed angina, coronary revascularisation, transient ischemic attack, or stroke. Preexisting cardiovascular disease was defined as a history of any one of these events, or of peripheral arterial disease, at baseline.

Time-to-event outcomes for all-cause mortality, cardiovascular mortality, and first fatal or nonfatal cardiovascular event were investigated using Cox proportional hazards models to derive hazard ratios (HR). Initial 2-stage IPD meta-analyses, modeling IAD for each study as both a continuous variable and serially as dichotomous variables with 1 mm Hg increments up to 20 mm Hg,^25^ were used to estimate heterogeneity. This was categorized according to *I*^2^ values as low (<25%) moderate (25%–50%) or high (>50%).^26^ All models were adjusted for baseline systolic BP, age, and sex.

One-stage random-effects flexible parametric models failed to converge (probably due to low between-study heterogeneity). Therefore, fixed-effect one-stage Cox proportional hazards modeling, stratified by study, was adopted, as per protocol.^22^ For prognostic modeling, 4 cohorts (4264 participants) were reserved as a validation dataset. Validation cohorts were chosen nonrandomly to include participants of both sexes covering the full age range.^27^ Two authors (F. Warren and C.E. Clark) independently chose 4 cohorts then agreed on the final selection by consensus (Figure S1).^28–31^ One-stage Cox proportional hazards models, stratified by study, used the remaining cohorts (the derivation dataset) to investigate effects of participant-level covariates on outcomes with inclusion of IAD within the model. Baseline HDL cholesterol was missing for 7 studies, so was omitted as a candidate covariate in model development. Statistically significant covariates (threshold *P*<0.1) were entered into multivariable models and retained if *P* values were <0.05. Excluded covariates were individually returned to check whether inclusion improved model fit, tested with likelihood ratio tests, and the Akaike Information Criterion.^32^

Final models were validated using calibration slopes and internal-external cross-validation analysis, with goodness of fit assessed using Harrell C statistic.^33^ Kaplan-Meier curves for derivation and validation cohorts were compared using cutoffs approximating to 10-year mortality risks groups of <10%, 10% to 20%, and >20%.

For participants without preexisting cardiovascular disease, systolic IAD was adjusted using Cox proportional hazards models stratified by study for 4 guideline-recommended cardiovascular risk scores: Atherosclerotic Cardiovascular Disease (ASCVD),^9,34^ Framingham,^8,35^ QRISK cardiovascular disease risk algorithm version 2 (QRISK2),^6,36^ and the European Systematic Coronary Risk Evaluation score (SCORE).^7,37^ Analyses were restricted to validated ethnicities and age ranges for each score (ASCVD: 40–79, Framingham: 20–79, QRISK2: 25–84, and SCORE: 40–65 years).^34–37^

Inclusion bias was assessed by comparing outcomes in the INTERPRESS-IPD dataset with published cohorts not in the Collaboration. Publication bias was assessed visually using a funnel plot and quantified with Egger test.^38^

Following multiple imputation of missing baseline data by chained equations, risk modeling was repeated and compared with models derived using observed data only; no missing outcome data were imputed. A total of 32 imputed datasets were produced, imputing baseline data across the pooled dataset, accounting for study in the imputation algorithm. A 3-person Public Advisory Group, facilitated by K. Boddy, was fully integrated throughout the study, contributing to bimonthly project meetings and facilitating dissemination of findings in accessible ways.

## Results

**Figure 1.**
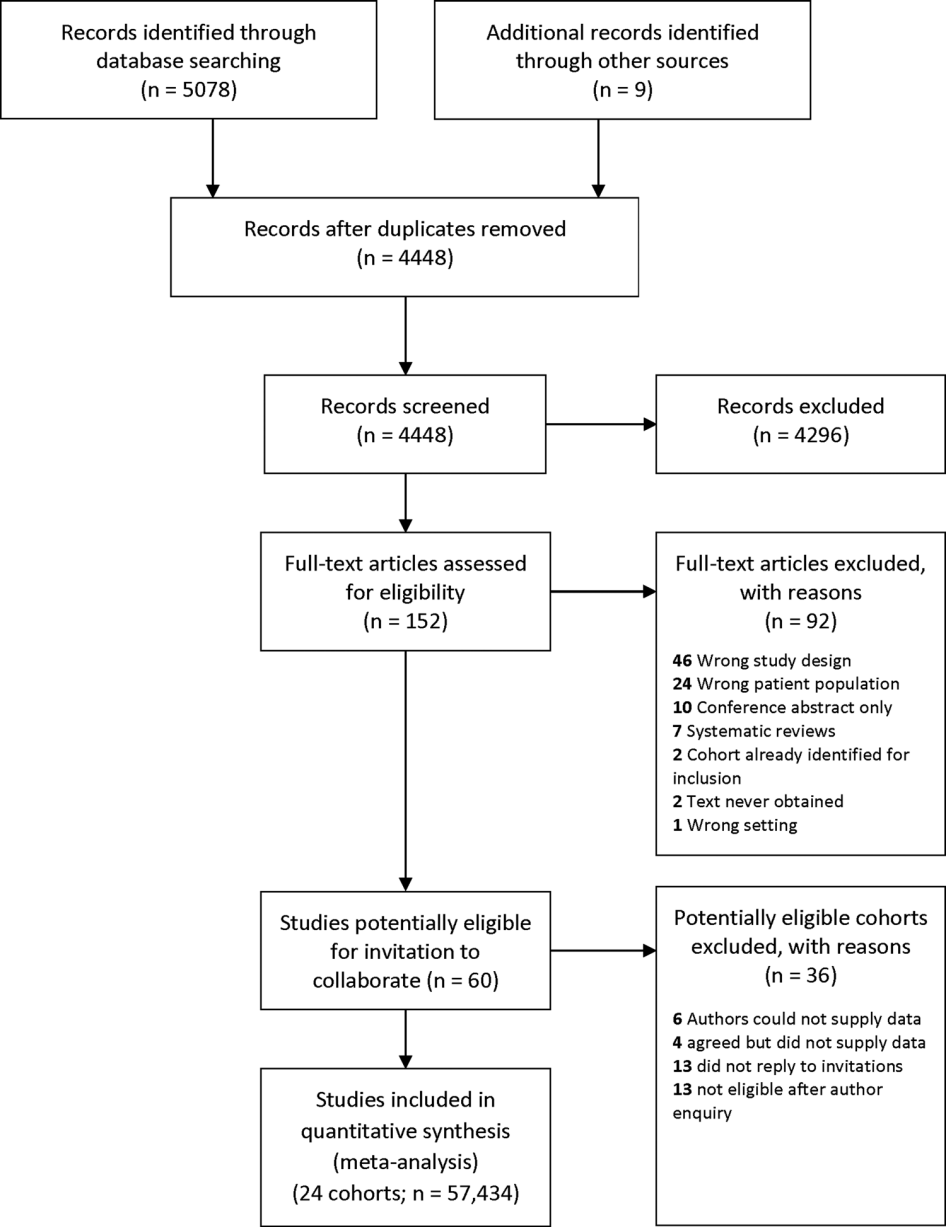
**PRISMA flow chart for literature search.**

Twenty-four studies contributed data to the Collaboration (Figure 1; Table S4); ten others were eligible but unable to supply data,^21,39–47^ and a further 26 were ruled out after consultations with authors (Table S5).^48–73^ Records for 53 827 (93.7%) participants contained at least one pair of BP readings. Mean age was 60.3 (SD, 12.5; range, 18–102), 47.8% were female, and baseline BP was 138.3/80.9 (SD, 21.8/11.8) mm Hg (Table S6). Studies originated from Western Europe (14), United States (7), East Asia (2), and Sub-Saharan Africa (1). Most participants were White (76%); other ethnicities were East Asian (8.6%), African American (6.0%), Hispanic American (3.8%) Black African (2.0%) with 0.8% unknown (coded as other; Table S2) and 2.8% missing. Studies recruited subjects from community or primary care registers (n=20),^16,19,28–31,74–87^ study recruitment clinics (n=3),^88–90^ and a Veterans’ medical service (n=1).^20^ Participants were free of cardiovascular disease in 3 cohorts,^11,28,74^ had diabetes in 2,^16,87^ hypertension in 1^19^ and were selected for intermediate vascular risk (10-year coronary event risk between 5 and 15%) in another.^86^ Remaining cohorts were not selected according to cardiovascular risk factors (Table S4). Distribution of variables and outcomes by study is described in the Tables S7 through S9).

### Study quality and assessment of bias

Modified Quality Improvement in Prognostic Studies assessments judged 19 datasets to be at low risk of bias; 5 were unclear and none high (Table S10); Quality Improvement in Prognostic Studies judgments were nondiscriminatory for outcomes. One published IAD study was not included in the INTERPRESS-IPD dataset; heterogeneity between HRs for it and included studies was low (*I*^2^=0%; Figure S1).^21^ We found no evidence of publication bias (Egger *P*=0.657; Figure S2).

### Time-to-Event Analyses and Model Development

#### All-Cause Mortality

All-cause mortality was reported in 23 studies with 4939 (9.2%) deaths (Figure S3). Two-stage random-effects modeling in 50 661 cases, adjusted for age, sex, and baseline systolic BP, identified that continuous IAD was significantly associated with time to all-cause mortality (HR, 1.04 [95% CI, 1.01–1.07] per 5 mm Hg of absolute IAD; heterogeneity between studies was moderate (*I*^2^=35%; Figure S4). Using 35 901 participants from 19 cohorts with complete data for candidate covariates as the derivation dataset systolic, IAD was a significant predictor of mortality (HR, 1.05 [1.02–1.08] per 5 mm Hg) with baseline systolic BP, age, sex, ethnicity, current smoking, total cholesterol, hypertensive, and diabetic status. Findings using all available data from those cohorts (40 124 participants) were similar (HR, 1.05 [1.02–1.07] per 5 mm Hg; Table 1). Harrell C statistic showed overlapping values between derivation (0.76 [95% CI, 0.75–0.77]) and validation (0.77 [95% CI, 0.75–0.80]) cohorts, with an overall pooled calibration slope on internal-external cross-validation of 0.97 (95% CI, 0.88–1.06]; Figure S5).

**Table 1.**
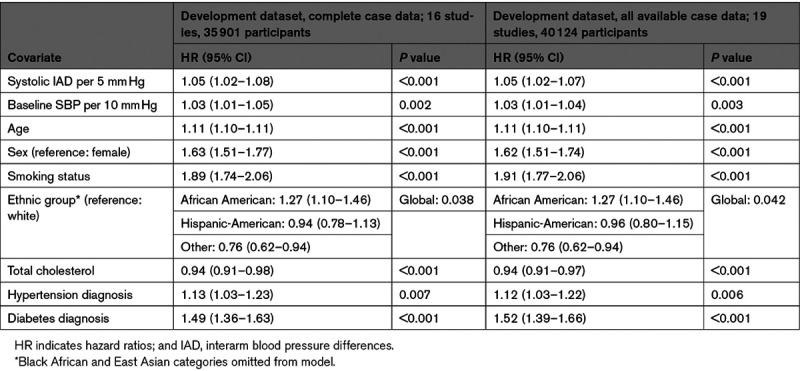
Results of One-Stage Meta-Analysis to Develop Overall Final Model for All-Cause Mortality

**Figure 2.**
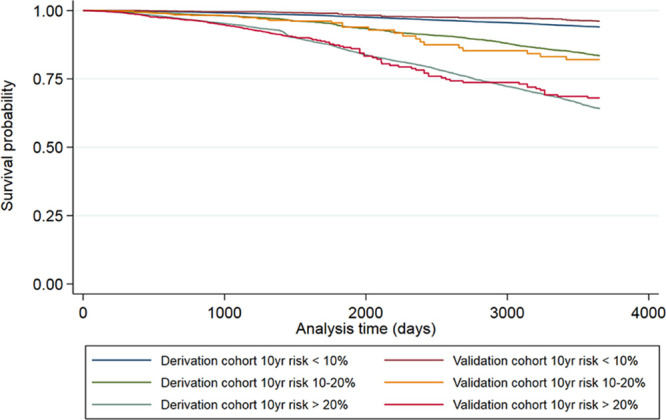
**Kaplan-Meier plots comparing all-cause mortality for the derivation and validation cohorts for 3 risk groups corresponding to 10% and 20% 10-year risk thresholds.**

**Figure 3.**
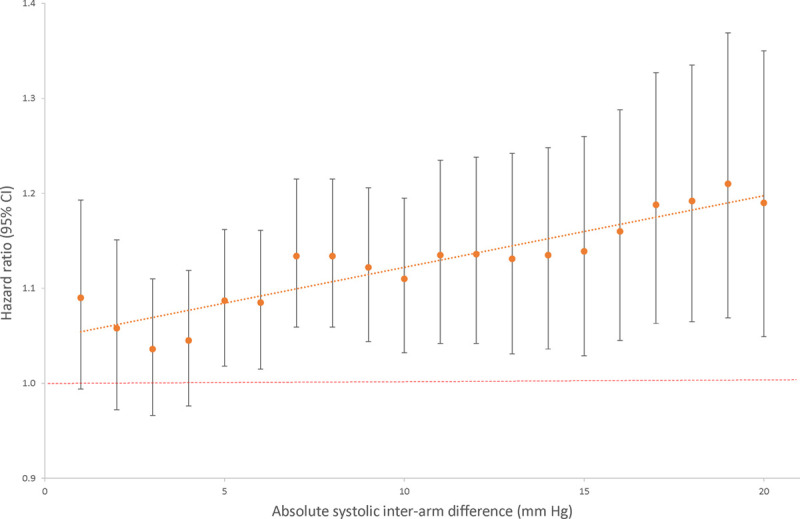
**All-cause mortality hazard ratios for systolic interarm difference in fully adjusted model.**

Hazard curves for each risk group showed good overlap between derivation and validation cohorts and good separation between groups (Figure 2). Using the model described above and all available data, dichotomized IAD cutoffs at 1 mm Hg increments indicated increasing hazards of all-cause mortality for an IAD rising from ≥5 mm Hg (HR for IAD ≥5 mm Hg: 1.07 [95% CI, 1.01–1.14]; *I*^2^=0%; 23 studies, 50 661 participants; Figure 3).

After imputation of missing baseline covariates (HDL, 7 studies; body mass index, 1 study), IAD was no longer a significant predictor of all-cause mortality in the fully adjusted (IAD plus all covariates found to be statistically significant) model (Table S11).

#### Cardiovascular Mortality

Cardiovascular deaths were reported for 1435 (3.0%) of 48 261 participants from 21 studies (Figure S3). Continuous systolic IAD, adjusted for age, sex, and baseline systolic BP was significantly associated with cardiovascular mortality (HR, 1.07 [95% CI, 1.03–1.12]; *I*^2^=11%) per 5 mm Hg of IAD (Figure S6). The derivation dataset for cardiovascular risk modeling consisted of 18 studies (33 650 participants with complete case data). The final model included systolic IAD (HR, 1.06 [95% CI, 1.02–1.11] per 5 mm Hg) and, apart from total cholesterol, the same coefficients as the all-cause mortality model (Table 2). Harrell C statistics were good and overlapped for the derivation (0.80 [95% CI, 0.79–0.82]) and validation (0.83 [95% CI, 0.79–0.87]) cohorts. Internal-external cross-validation gave a pooled calibration slope of 0.97 (95% CI, 0.83–1.10; Figure S7). Continuous systolic IAD remained a significant predictor of cardiovascular mortality after imputation of missing covariates (Table S12).

**Table 2.**
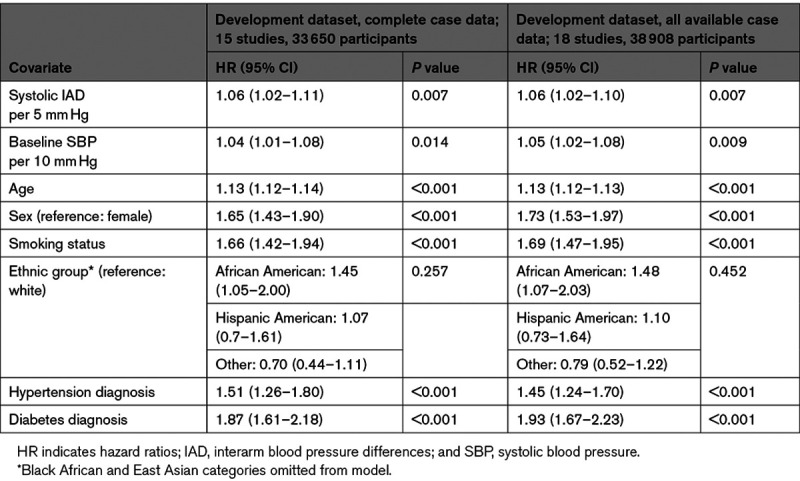
Results of One-Stage Meta-Analysis to Develop Overall Final Model for Cardiovascular Mortality

#### Fatal and Nonfatal Cardiovascular Events

Of 51 405 participants in 23 studies, 5800 (11.3%) had an event within 10 years. In the 2-stage random-effects model, continuous systolic IAD was associated with greater risk for any cardiovascular event after adjustment for age, sex, and baseline systolic BP (HR, 1.03 per 5 mm Hg increment [95% CI, 1.00–1.06]; *I*^2^=25%; Figure S8). In the 1-stage fixed-effect model, continuous systolic IAD was not associated with risk of events (HR, 1.01 per 5 mm Hg increment [95% CI, 0.99–1.03], *P*=0.174); therefore, multivariable modeling was not pursued as per protocol. After imputation of missing data, however, systolic IAD became a significant predictor of outcome (Table S13).

### Interarm Difference and Existing Cardiovascular Risk Models

Systolic IADs remained significantly associated with fatal or nonfatal cardiovascular events over 10 years after adjustment for the ASCVD, Framingham, or QRISK2 risk scores: residual HRs for an IAD ≥10 mm Hg ranged from 1.2 to 1.4 (Table 3). As continuous variables, HRs per 5 mm Hg increment of systolic IAD was 1.04 (95% CI, 1.01–1.08) when adjusted for the Framingham score, 1.04 (95% CI, 1.00–1.08) for ASCVD and 1.12 (95% CI, 1.06–1.18) for QRISK2, representing 4% to 12% increases in 10-year cardiovascular risk scores (Figure S9). After restricting analyses to a cohort eligible by age for all 3 risk scores (40–79 years), variation in HRs between scores was minimized (Table 3). The HR of IAD for cardiovascular mortality, after adjustment for SCORE in 18 239 participants was not statistically significant (HR, 1.07 per 5 mm Hg increment [95% CI, 0.97–1.18]; *P*=0.178).

**Table 3.**
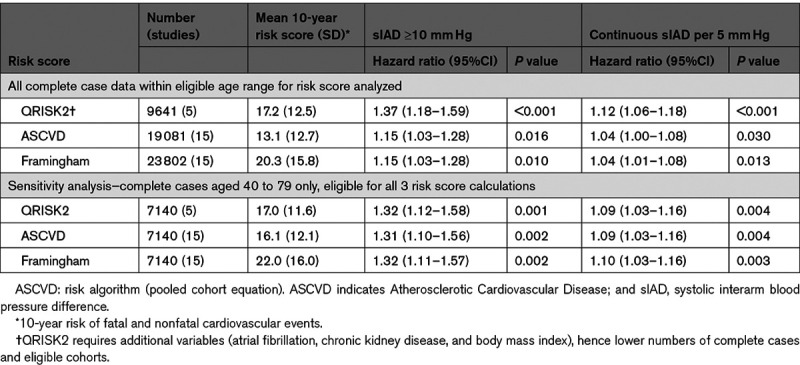
Hazard Ratios for First Cardiovascular Fatal and Nonfatal Events for Interarm Differences Adjusted for 10-Year Cardiovascular Risk Scores

## Discussion

This is the first international IPD meta-analysis to report and model the risks of all-cause mortality, cardiovascular mortality, and fatal or nonfatal cardiovascular events associated with a systolic IAD. We have developed and validated multivariable risk models incorporating IAD to predict all-cause and cardiovascular mortality and demonstrated that IAD remains associated with additional risk of cardiovascular events after adjustment for 3 currently used cardiovascular event risk prediction scores.

People with an IAD ≥5 mm Hg had higher mortality rates than those with an IAD <5 mm Hg. Each 1 mm Hg increment in IAD was associated with approximately a 1% inflation of a given 10-year risk for all-cause mortality and a 1% to 2% inflation of 10-year risk of cardiovascular death. These prospective findings are based on a single pair of BP measurements, the most readily available means of measuring IAD, and so are of direct relevance to current clinical practice.

Previous studies have generally failed to observe a dose-response association of rising HRs for mortality with increasing IADs, as now demonstrated after adjustment in this IPD analysis.^12,18,74^ This supports the case for IAD as a marker of cardiovascular risk.^91^ Our findings confirm the risk associated with IAD after adjusting for Framingham risk scores and, for the first time, also show this additional cardiovascular risk after adjustment for ASCVD or QRISK2 scores in patients without cardiovascular disease at baseline.^19–21^ This suggests that the additional impact of IAD on risk should be considered when assessing cardiovascular risk using existing risk equations. This approach is consistent with guidelines to recognize additional individual cardiovascular risk factors that are not incorporated in risk scores.^7^ IAD was not a significant predictor of cardiovascular mortality after adjustment for SCORE. Gradual improvements in secondary prevention mean fewer cardiovascular deaths now occur as a proportion of total events, rendering prediction of fatal events alone less clinically relevant. The cardiovascular death rate over 10 years in SCORE eligible participants in this study was only 192 out of 20 785 (0.9%) in comparison to 7934 out of 205 178 (3.9%) in the original SCORE derivation study.^92^

Current European and National Institute for Health and Care Excellence guidance note that a systolic IAD >15 mm Hg is associated with additional cardiovascular risk.^6,7^ Our findings confirm previous individual cohort study findings of risk arising for IADs from the lower threshold of ≥ 5 mm Hg.^20,74,89^ Sequential measurement of arms can overestimate magnitude of IADs compared with simultaneous measurement but also has a high negative predictive value in ruling out a simultaneous IAD.^16,93^ Taking a conservative view of our findings, we think that a 10 mm Hg difference can now be reasonably regarded as an upper limit of normal for systolic IAD measured sequentially during routine assessment. This information should be incorporated into future guidelines and clinical practice in assessing cardiovascular risk.

Guidelines advise checking BP in both arms when assessing people for hypertension but are widely ignored.^6,7,94^ Failure to check both arms risks errors in BP interpretation and management and suboptimal BP control.^95,96^ Uptake of bilateral arm BP measurement is increasing but has not yet become standard care.^97^ Our findings show how IAD identifies additional cardiovascular risk beyond ASCVD, Framingham, or QRISK2 scores alone. This could inform decision-making with individuals about primary prevention of cardiovascular disease. These results are based on IAD derived from a single sequential pair of BP measurements, easily obtained in practice, and challenge advice that IAD should be confirmed using simultaneous measurement during clinical assessment.^7^

### Study Limitations

Comprehensive searches were undertaken for cohorts that reported either IAD or ankle-brachial index. Additional data were identified through author contacts.^77,81,82,88,89^ In total, we combined data from 24 of 34 confirmed eligible studies, representing two-thirds of potentially eligible participants, and comparison with the one published eligible IAD study that we did not obtain showed no evidence of inclusion bias. These IPD meta-analyses have required considerable data cleaning and preparation; closure of data inclusion in 2017 means that we could have excluded relevant recent publications, however, continued search updates to April 2020 have only identified one new potentially eligible study.^98^ Our search strategy could have missed cohorts or registries holding bilateral BP data if no linked IAD or ankle-brachial index publications exist. We did not search specifically for pulse-wave velocity studies but are aware that recent technology for measuring this can generate bilateral BP data.^99^

We derived and validated risk models for all-cause and cardiovascular mortality, which, in addition to IAD, included risk markers congruent with previously published risk models.^100^ Participants were largely based not only in North America and low risk (according to SCORE definition) European countries^37^ but also included Asia and sub-Saharan Africa, thus offering relevance to a range of populations. Since residual confounding with existing risk scores may make it easier to show that a new marker improves prediction, we regard our de novo derived risk models as key demonstrations of the contribution of IAD to risk prediction.^101^ The amount of missing data varied widely across studies and across variables. Sensitivity analyses incorporating multiple imputation of missing data were carried out on the assumption that missing data are missing at random. Some discrepancies between results of models using observed data alone and models using observed and imputed data were noted, suggesting that the missing at random assumption may not be valid; however, we emphasize that the models using observed data only were our primary outcomes.

No intervention studies based on detection of an IAD exist, and it is vital that novel risk markers are fully evaluated.^102^ In general, there is a paucity of evidence that interventions based on cardiovascular risk result in reductions in mortality.^103^ This study has incorporated IAD into multivariable risk models but these will need prospective evaluation.

### Conclusions

This study demonstrates and quantifies the independent association of IAD with cardiovascular events and mortality. Findings based on a single pair of sequential BP readings suggest a reduction of the currently accepted normal systolic IAD to a limit of 10 mm Hg can be justified. These findings are relevant to primary care practice and to low resource settings. BP should be measured in both arms when undertaking cardiovascular assessments.

### Perspectives

Associations of sequentially measured systolic interarm differences in BP with all-cause mortality, cardiovascular mortality, and events were investigated in a multinational individual participant data meta-analyses including 53 827 participants. Adjusted associations of IAD with all-cause mortality and cardiovascular mortality were confirmed, and models incorporating IAD were developed and validated. Interarm differences were also associated with higher risks of cardiovascular events after adjustment for commonly used cardiovascular risk scores. BP should be measured in both arms during cardiovascular assessment. Further research is needed to determine whether selection of intensive cardiovascular risk reduction strategies, on the basis of a detected interarm difference, can modify outcomes for people with an interarm difference.

## Acknowledgments

We thank the following for their involvement in the Inter-arm Blood Pressure Difference - Individual Participant Data (INTERPRESS-IPD) Collaboration: Independent Monitoring Group: Alun Hughes (chair), Gary Collins, and Tom Fahey. Data management Group: Tim Eames and Sofia Sanabria (Exeter Clinical Trials Unit). Public Advisory Group: J. Goddard, M. Turner, and N. Reed. Ellie Kingsland provided administrative support throughout the study. We thank Professor Gerry Fowkes (ankle-brachial index collaboration) and Professor Colin Baigent (Antithrombotic Trialists’ and Cholesterol Treatment Trialists’) for their assistance in establishing that existing triallists’ collaborations could not address these research questions. Morwenna Rogers assisted in development and piloting of the search strategy, Francesco Cappuccio (European Society of Hypertension Working Group on Hypertension and Cardiovascular Risk assessment in subjects living in or emigrating from Low Resource Settings) advised on ethnic classification across cohorts. Oana Builete assisted with translation of Spanish language papers. Chang-Sheng Sheng assisted in preparing the Elderly Chinese Cohort data. James White piloted and commented on the draft data request proforma. We thank Stephen Hippisley-Cox at ClinRisk Ltd for calculating QRISK2-2017 scores from an input data file prepared by the authors. C.E. Clark proposed the study; C.E. Clark, K. Boddy, F.C. Warren, V. Aboyans, L. Cloutier, R.J. McManus, A.C. Shore, R.S. Taylor, and J.L. Campbell were co-applicants on this project; S.T.J. McDonagh and S.F. Moore were researchers on the project. The protocol was drafted by C.E. Clark, F.C. Warren, R.S. Taylor, and J.L. Campbell, then revised, and edited by all co-applicants. K. Boddy led and coordinated public and patient involvement throughout the study; J. Goddard, N. Reed, M. Turner were the public and patient advisors, S.T.J. McDonagh, S.F. Moore, J.L. Campbell, A.C. Shore, and C.E. Clark undertook literature searches, C.E. Clark liaised with and obtained data from collaborating authors. F.C. Warren undertook data cleaning and led the analyses, R.S. Taylor supervised the analyses, A.C. Shore, L. Cloutier, R.J. McManus, and V. Aboyans advised on study conduct, analysis, and interpretation of findings. All authors contributed to the article and have read and reviewed the final article, which was then approved by the independent monitoring group. C.E. Clark has full access to the data and will act as guarantor for the study. The following collaborating authors contributed data to the INTERPRESS-IPD Collaboration and contributed to the final article: Mid Devon cohorts: C.E. Clark; Elderly Chinese: J.-G. Wang; Vietnam Experience Study: J. White; Diabetes Alliance for Research in England: A. Hattersley; Aspirin for Asymptomatic Atherosclerosis: J. Price; Invecchiare in Chianti: L. Ferrucci; Heinz-Nixdorf Recall Study: R. Erbel; Second Manifestations of ARTerial disease: J. Westerink; LRC & San Diego Population Study: M. Criqui; Fuencarral Health Center: C. Lahoz; Peripheral Arterial Disease: M.T. Alzamora; Epidemiology of dementia in Central Africa: M. Guerchet; Multi Ethnic Study of Atherosclerosis: R.L. McClelland; Lifestyle Interventions and Independence for Elders & Walking and Leg Circulation Study: M.M. McDermott; Limburg Peripheral Arterial Occlusive Disease Study: H.E. Stoffers; Men born in 1914: G. Engström; Look Action for Health in Diabetes: M. Espeland; Kinmen Health Survey: S.-Y. Chuang; Viborg Women Cohort: M. Dahl; Surrogate markers for Micro- and Macrovascular hard endpoints as Innovative diabetes tools: A.C. Shore; Improving interMediAte RisK management Study: R. Ramos Blanes.

## Sources of Funding

This study was funded by the National Institute for Health Research (NIHR) Research for Patient Benefit Programme (PB-PG-0215-36009). C.E. Clark was supported by an NIHR Clinical Lectureship award. R.J. McManus receives support from the NIHR Oxford Collaboration for Leadership in Applied Health Research and Care. The views expressed are those of the authors and not necessarily those of the NIHR, the National Health Service, or the Department of Health.

## Disclosures

C.E. Clark has received an honorarium from Bayer (unrelated to interarm BP differences work) and has been loaned bilateral blood pressure (BP) monitors by Microlife and Jawon Medical for unrestricted evaluation. R.J. McManus has received monitors for BP research from Omron. No company has had, or will have, any involvement in the design, conduct or reporting of this study. The other authors report no conflicts.

## Supplementary Material



## Novelty and Significance

### What Is New?

- To determine the impact of differences in blood pressure between arms on cardiovascular outcomes (eg, heart attack, death), data from over 57 000 individuals from 24 studies around the world were collated into one large dataset and analyzed.
- Results suggest that interarm differences above 5 mm of mercury (mm Hg) predict all-cause mortality, cardiovascular mortality and cardiovascular events.
- Results are based on a single pair of measurements with blood pressure measured in one arm and then the other, and challenge advice that interarm differences in blood pressure should be confirmed using a simultaneous method (blood pressure measured in both arms at the same time).

### What Is Relevant?

- Interarm differences in blood pressure identify additional risk of having a cardiovascular event beyond that predicted by existing cardiovascular risk scores alone; this could inform clinical decisions regarding primary prevention of cardiovascular disease.

### Summary

A 10 mm Hg difference in systolic blood pressure between arms should now be regarded as an upper limit of normal. Checking blood pressure in both arms, beyond choosing which arm to measure for the detection and management of high blood pressure, is a key part of cardiovascular risk assessment and should become routine clinical practice.
