# Supplementary material for: Associations Between Systolic Interarm Differences in Blood Pressure and Cardiovascular Disease Outcomes and Mortality: Individual Participant Data Meta-Analysis, Development and Validation of a Prognostic Algorithm: The INTERPRESS-IPD Collaboration
Source: Hypertension. 2020 Dec 21;77(2):650–61. doi: 10.1161/HYPERTENSIONAHA.120.15997 (PMC7803446; doi:10.1161/HYPERTENSIONAHA.120.15997)
Supplement: Supplementary file 1 [file hyp-77-650-s001.docx]

**Online data supplement**

**­Associations between systolic inter-arm differences in blood pressure and cardiovascular disease outcomes and mortality**

Christopher E Clark PhD MBChB FRCP^1^, Fiona C Warren PhD^1^, Kate Boddy MSc^2^, Sinead TJ McDonagh PhD^1^, Sarah F Moore MB BChir^1^, John Goddard^2^, Nigel Reed^2^, Malcolm Turner^2^, Maria Teresa Alzamora PhD^3^, Rafel Ramos Blanes MD PhD^4^, Shao-Yuan Chuang PhD^5^, Michael Criqui MD, MPH^6^, Marie Dahl RN PhD^7^, Gunnar Engström MD PhD^8^, Raimund Erbel MD^9^, Mark Espeland PhD^10^, Luigi Ferrucci MD, PhD ^11^, Maëlenn Guerchet PhD^12^, Andrew Hattersley DM^13^, Carlos Lahoz MD PhD^14^, Robyn L McClelland PhD^15^, Mary M McDermott MD^16^, Jackie Price MD^17^, Henri E Stoffers MD PhD ^18^, Ji-Guang Wang MD PhD^19^, Jan Westerink MD PhD^20^, James White PhD^21^, Lyne Cloutier RN PhD^22^, Rod S Taylor PhD^1 &23^, Angela C Shore PhD^24^, Richard J McManus PhD MBBS FRCGP^25^, Victor Aboyans MD PhD FAHA ^12,& 26^, John L Campbell MD MBChB FRCGP^1^

1. Primary Care Research Group, Institute of Health Services Research, University of Exeter Medical School, College of Medicine & Health, Smeall Building, St Luke’s Campus, Magdalen Road, Exeter, Devon, England, EX1 2LU

2. Patient and Public Involvement Team, PenCLAHRC, University of Exeter Medical School, College of Medicine & Health, South Cloisters, St Luke's Campus, Magdalen Road, Exeter, Devon, England, EX1 2LU

3. Unitat de Suport a la Recerca Metropolitana Nord, Fundació Institut Universitari per a la recerca a l'Atenció Primària de Salut Jordi Gol i Gurina (IDIAPJGol), Mataró, Spain

4. Unitat de Suport a la Recerca Girona. Fundació Institut Universitari per a la recerca a l'Atenció Primària de Salut Jordi Gol i Gurina (IDIAPJGol), Institut d’Investigació Biomèdica de Girona (IdIBGi), Department of Medical Sciences, School of Medicine, University of Girona, Girona, Spain

5. Institute of Population Health Sciences, National Health Research Institutes (NHRI), No35. Keyan Road, Zhunan, Miaoli County 35053, Taiwan, R.O.C

6. Department of Family Medicine and Public Health, University of California, San Diego, School of Medicine, 9500 Gilman Drive, La Jolla, CA, 92093-0628, USA

7. Vascular Research Unit, Department of Vascular Surgery, Viborg Regional Hospital, Heibergs Allé 4, 8800 Viborg, Denmark and Department of Clinical Medicine, Aarhus University, Denmark

8. Department of Clinical Science in Malmö, Lund University, CRC 60:13, Box 50332, 20213 Malmö, Sweden

9. Institute of Medical Informatics, Biometry and Epidemiology, University Hospital Essen, Hufelandstraße 55, D-45147 Essen, Germany

10. Wake Forest School of Medicine, North Carolina, USA

11. National Institute on Aging, 251 Bayview Boulevard, Baltimore MD 21224, USA

12. INSERM U1094 & IRD, Tropical Neuroepidemiology, Institut d'Epidémiologie et de Neurologie Tropicale (IENT), Faculté de Médecine de l'Université de Limoges - 2 rue du Dr Marcland - 87 025 Limoges Cedex, France

13. Institute of Biomedical and Clinical Science, University of Exeter Medical School, College of Medicine and Health, RILD, Barrack Road, Exeter, Devon, England, EX2 5DW

14. Lípid and Vascular Risk Unit. Internal Medicine Service. Carlos III - La Paz Hospital. Madrid. Spain.

15. Department of Biostatistics, University of Washington, Washington, USA

16. Northwestern University Feinberg School of Medicine, 750 North Lake Shore Drive, 10^th^ floor, Chicago, IL b60611, USA

17. Usher Institute of Population Health Sciences and Informatics, University of Edinburgh, Scotland, EH8 9AG

18 Department of Family Medicine, CAPHRI Care and Public Health Research Institute, Maastricht University, P.O.Box 616, 6200 MD Maastricht, The Netherlands

19. Centre for Epidemiological Studies and Clinical Trials, Shanghai Key Laboratory of Hypertension, The Shanghai Institute of Hypertension, Ruijin Hospital, Shanghai Jiaotong University School of Medicine, Ruijin 2nd Road 197, Shanghai 200025, China

20. Department of Vascular Medicine, University Medical Center Utrecht, Utrecht, The Netherlands

21. DECIPHer, Centre for Trials Research, College of Biomedical and Life Sciences, Cardiff University, 4th Floor, Heath Park, Cardiff, CF14 4YS

22. Département des sciences infirmières, Université du Québec à Trois-Rivières, 3351 Boulevard des Forges, Trois-Rivières, Québec, Canada, G9A5H7

23. MRC/CSO Social and Public Health Sciences Unit & Robertson Centre for Biostatistics, Institute of Health and Well Being, University of Glasgow

24. NIHR Exeter Clinical Research Facility, Royal Devon and Exeter Hospital and University of Exeter College of Medicine & Health, Barrack Road, Exeter, Devon, England, EX2 5AX

25. Nuffield Department of Primary Care Health Sciences, University of Oxford, Radcliffe Primary Care Building, Radcliffe Observatory Quarter, Woodstock Road, Oxford, England, OX2 6GG

26. Department of Cardiology, Dupuytren University Hospital, and Inserm 1094,
Tropical Neuroepidemiology, Limoges, France

# List of supplemental materials

Table S1 – Search strategy

Table S2 – Summary of consensus ethnicity classifications for studies

Table S3 – Quality assessment – modified QUIPS tool

Table S4 – Characteristics of included studies

Table S5 – Potentially eligible cohorts excluded with reasons

Table S6 - Descriptive pooled baseline data from all studies

Table S7 - Study level outcomes and attrition, all participants

Table S8 - Study level baseline demographic and health characteristics

Table S9 - Distribution of morbidities at baseline

Table S10 - Modified QUIPS judgements for included studies

Table S11 - Results of one-stage meta-analysis of observed plus imputed participant baseline data for all-cause mortality: hazard ratios for model including all selected covariates

Table S12 - Results of one-stage meta-analysis of observed plus imputed data for cardiovascular mortality: hazard ratios for model including all selected covariates

Table S13 - Results of one-stage meta-analysis of observed plus imputed data for cardiovascular fatal and non-fatal events: hazard ratios for model including all selected covariates

Figure S1 – Forest plot of cardiovascular mortality hazard ratios for included and non-included studies

Figure S2 - Funnel plot derived from the two-stage all-cause mortality model in all 23 studies including continuous systolic inter-arm difference as the only covariate

Figure S3 - Contributions of cohorts to individual analyses

Figure S4 - Forest plot of continuous systolic inter-arm difference, per 5mmHg increment, with adjustment for baseline systolic BP, age and sex: time to all-cause mortality

Figure S5 - Random effects meta-analysis of calibration slope for all-cause mortality

Figure S6 - Forest plot of continuous systolic inter-arm difference, per 5mmHg increment, with adjustment for baseline systolic BP, age and sex: time to cardiovascular mortality

Figure S7 - Random effects meta-analysis of calibration slope for cardiovascular mortality

Figure S8 - Forest plot of cardiovascular fatal or non-fatal first events within 10 years for continuous systolic inter-arm difference, per 5mmHg increment, with adjustment for baseline systolic BP, age and sex

Figure S9 - Modified ten-year risk score tables showing adjusted risk scores according to systolic inter-arm difference

# Supplemental references

1. Clark CE, Taylor RS, Shore AC, Campbell JL. The difference in blood pressure readings between arms and survival: primary care cohort study. *BMJ*. 2012;344:e1327

2. Clark CE, Powell RJ. The differential blood pressure sign in general practice: prevalence and prognostic value. *Family Practice*. 2002;19:439-441

3. Sheng CS, Liu M, Zeng WF, Huang QF, Li Y, Wang JG. Four-Limb Blood Pressure as Predictors of Mortality in Elderly Chinese. *Hypertension*. 2013;61:1155-1160

4. White J, Mortensen LH, Kivimaki M, Gale CR, Batty GD. Interarm differences in systolic blood pressure and mortality among US army veterans: aetiological associations and risk prediction in the Vietnam experience study. *Eur.J.Prev.Cardiol.* 2014;21:1394-1400

5. Clark CE, Steele AM, Taylor RS, Shore AC, Ukoumunne OC, Campbell JL. Inter-arm blood pressure difference in people with diabetes: measurement and vascular and mortality implications: a cohort study. *Diabetes Care*. 2014;37:1-8

6. Clark CE, Taylor RS, Butcher I, Stewart MC, Price J, Fowkes FGR, Shore AC, Campbell JL. Inter-arm blood pressure difference and mortality: a cohort study in an asymptomatic primary care population at elevated cardiovascular risk. *British Journal of General Practice*. 2016;66:241-242

7. Clark CE, Thomas D, Llewellyn DJ, Ferrucci L, Bandinelli S, Campbell JL. Systolic inter-arm blood pressure difference and risk of cognitive decline in older people: a cohort study. *British Journal of General Practice*. 2020;70:e472-e480

8. Erbel R, Mohlenkamp S, Moebus S, Schmermund A, Lehmann N, Stang A, Dragano N, Gronemeyer D, Seibel R, Kalsch H, Brocker-Preuss M, Mann K, Siegrist J, Jockel KH, Heinz Nixdorf Recall Study Investigative G. Coronary risk stratification, discrimination, and reclassification improvement based on quantification of subclinical coronary atherosclerosis: the Heinz Nixdorf Recall study. *J Am Coll Cardiol*. 2010;56:1397-1406

9. Kranenburg G, Spiering W, de Jong PA, Kappelle LJ, de Borst GJ, Cramer MJ, Visseren FLJ, Aboyans V, Westerink J. Inter-arm systolic blood pressure differences, relations with future vascular events and mortality in patients with and without manifest vascular disease. *International journal of cardiology*. 2017;244:271-276

10. Criqui MH, Fronek A, Barrett-Connor E, Klauber MR, Gabriel S, Goodman D. The prevalence of peripheral arterial disease in a defined population. *Circulation*. 1985;71:510-515

11. Wassel CL, Loomba R, Ix JH, Allison MA, Denenberg JO, Criqui MH. Family History of Peripheral Artery Disease is associated with Prevalence and Severity of Peripheral Artery Disease: The San Diego Population Study (SDPS). *Journal of the American College of Cardiology*. 2011;58:1386-1392

12. Lahoz C, Barrionuevo M, Garcia-Fernandez T, Vicente I, Garcia-Iglesias MF, Mostaza JM. Cardiovascular morbidity-mortality associated to ankle-brachial index in the general population. [Spanish]. *Revista Clinica Espanola*. 2014;214:1-7

13. Alzamora MT, Baena-Díez JM, Sorribes M, Forés R, Toran P, Vicheto M, Pera G, Reina MD, Albaladejo C, Llussà J, Bundó M, Sancho A, Heras A, Rubiés J, Arenillas JF. Peripheral Arterial Disease Study (PERART): Prevalence and predictive values of asymptomatic peripheral arterial occlusive disease related to cardiovascular morbidity and mortality. *BMC public health*. 2007;7:1-7

14. Guerchet M, Mbelesso P, Ndamba-Bandzouzi B, Pilleron S, Desormais I, Lacroix P, Aboyans V, Jesus P, Desport JC, Tchalla AE, Marin B, Lambert JC, Clement JP, Dartigues JF, Preux PM. Epidemiology of dementia in Central Africa (EPIDEMCA): protocol for a multicentre population-based study in rural and urban areas of the Central African Republic and the Republic of Congo. *SpringerPlus*. 2014;3:338

15. Bild DE, Bluemke DA, Burke GL, Detrano R, ez Roux AV, Folsom AR, Greenland P, David R, Kronmal R, Liu K, et al. Multi-Ethnic Study of Atherosclerosis: Objectives and Design. *American Journal of Epidemiology*. 2002;156:871-881

16. Espeland MA, Newman AB, Sink K, Gill TM, King AC, Miller ME, Guralnik J, Katula J, Church T, Manini T, et al. Associations Between Ankle-Brachial Index and Cognitive Function: Results From the Lifestyle Interventions and Independence for Elders Trial. *Journal of the American Medical Directors Association*. 2015;16:682-689

17. Hooi JD, Stoffers HE, Kester AD, Rinkens PE, Kaiser V, van Ree JW, Knottnerus JA. Risk factors and cardiovascular diseases associated with asymptomatic peripheral arterial occlusive disease: The Limburg PAOD Study. *Scand J Prim Health Care*. 1998;16:177-182

18. Ogren M, Hedblad B, Engstrom G, Janzon L. Prevalence and prognostic significance of asymptomatic peripheral arterial disease in 68-year-old men with diabetes. Results from the population study 'Men born in 1914' from Malmo, Sweden. *European Journal of Vascular & Endovascular Surgery*. 2005;29:182-189

19. Espeland MA, Beavers KM, Gibbs BB, Johnson KC, Hughes TM, Baker LD, Jakicic J, Korytkowski M, Miller M, Bray GA. Ankle-brachial index and inter-artery blood pressure differences as predictors of cognitive function in overweight and obese older adults with diabetes: Results from the Action for Health in Diabetes movement and memory study. *International Journal of Geriatric Psychiatry*. 2015;30:999-1007

20. Chuang SY, Sung SH, Cheng HM, Hsu PF, Chou P, Chen CH. Ankle-brachial index and brachial-ankle pulse wave velocity jointed to predict mortality in a community study. *European Heart Journal*. 2015;36:1015-1016

21. McGrae McDermott M, Greenland P, Liu K, et al. Leg symptoms in peripheral arterial disease: Associated clinical characteristics and functional impairment. *JAMA*. 2001;286:1599-1606

22. Dahl M, Frost L, Søgaard R, Klausen IC, Lorentzen V, Lindholt J. A population-based screening study for cardiovascular diseases and diabetes in Danish postmenopausal women: acceptability and prevalence. *BMC cardiovascular disorders*. 2018;18:20

23. Clark CE, Casanova F, Gooding K, Pamphilon N, Aizawa K, Mawson D, Adingupu D, Ball C, Worthington F, Elyas S, Strain WD, Gates PE, Shore AC, Campbell JL. Inter-arm blood pressure difference and arterial stiffness. *Journal of Hypertension*. 2014;32:e30

24. Marti R, Garcia-Regalado N, Garcia-Gil M, Marti R, Parramon D, Garcia-Ortiz L, Rigo F, Gomez-Marcos MA, Recio-Rodriguez JI, Garcia-Gil M, Ponjoan A, Quesada M, Ramos R. Improving interMediAte risk management. MARK study. *BMC Cardiovascular Disorders*. 2011;11:61

25. Shock NW, Gerontology Research Center (U.S.). *Normal human aging : the Baltimore longitudinal study of aging*. Washington, D.C.: U.S. Dept. of Health and Human Services; 1984.

26. Diehm C, Schuster A, Spengel FA, Trampisch HJ, Allenberg JR, Darius H, Haberl R, Fricke R, Holland-Letz T, Lange S, et al. getABI: German epidemiological trial on ankle brachial index for elderly patients in family practice to dedect peripheral arterial disease, significant marker for high mortality. *Vasa - Journal of Vascular Diseases*. 2002;31:241-248

27. Jager A, Kostense PJ, Ruhe HG, Heine RJ, Nijpels G, Dekker JM, Bouter LM, Stehouwer CD. Microalbuminuria and peripheral arterial disease are independent predictors of cardiovascular and all-cause mortality, especially among hypertensive subjects: five-year follow-up of the Hoorn Study. *Arteriosclerosis, Thrombosis & Vascular Biology*. 1999;19:617-624

28. Ortiz-Panozo E, Lajous M, Yunes-Diaz E, Mercado N, Luviano A, Flores-Silva F, Dircio-Delgado V, Cantu-Brito C, Catzin-Kuhlmann A, Lopez-Ridaura R. Peripheral arterial disease and carotid atherosclerosis among middle-aged women living in chiapas, mexico: Preliminary results. *Circulation. Conference: American Heart Association's Epidemiology and Prevention/Physical Activity, Nutrition and Metabolism*. 2013;127

29. Weinberg I, Gona P, O'Donnell CJ, Jaff MR, Murabito JM. The systolic blood pressure difference between arms and cardiovascular disease in the Framingham Heart Study. *American Journal of Medicine*. 2014;127:209-215

30. Kojima I, Ninomiya T, Hata J, Fukuhara M, Hirakawa Y, Mukai N, Yoshida D, Kitazono T, Kiyohara Y. A low ankle brachial index is associated with an increased risk of cardiovascular disease: the Hisayama study. *Journal of Atherosclerosis & Thrombosis*. 2014;21:966-973

31. De Graauw J, Chonchol M, Poppert H, Etgen T, Sander D. Relationship between kidney function and risk of asymptomatic peripheral arterial disease in elderly subjects. *Nephrology Dialysis Transplantation*. 2011;26:927-932

32. Allison MA, Laughlin GA, Barrett-Connor E, Langer R. Association between the ankle-brachial index and future coronary calcium (the Rancho Bernardo study). *American Journal of Cardiology*. 2006;97:181-186

33. Sakamoto R, Okumiya K, Ishine M, Wada T, Fujisawa M, Imai H, Ishimoto Y, Kimura Y, Fukutomi E, Chen W, Sasiwongsaroj K, Kato E, Otsuka K, Matsubayashi K. Predictors of difficulty in carrying out basic activities of daily living among the old-old: A 2-year community-based cohort study. *Geriatrics & gerontology international*. 2016;16:214-222

34. McKenna M, Wolfson S, Kuller L. The ratio of ankle and arm arterial pressure as an independent predictor of mortality. *Atherosclerosis*. 1991;87:119-128

35. Abbott RD, Petrovitch H, Rodriguez BL, Yano K, Schatz IJ, Popper JS, Masaki KH, Ross GW, Curb JD. Ankle/brachial blood pressure in men >70 years of age and the risk of coronary heart disease. *American Journal of Cardiology*. 2000;86:280-284

36. Carbayo Herencia JA, Artigao Rodenas LM, Divison Garrote JA, Caldevilla Bernardo D, Sanchis Domenech C, Torres Moreno P. Ankle-brachial index and the incidence of all-cause mortality and cardiovascular morbidity in a prospective cohort study of a general population. [Spanish]. *Clinica e Investigacion en Arteriosclerosis*. 2011;23:21-28

37. Filippella M, Lillaz E, Ciccarelli A, Giardina S, Massimetti E, Navaretta F, Antico A, Veronesi M, Lombardi G, Colao A, Ghigo E, Benso A, Doveri G. Ankle brachial pressure index usefulness as predictor factor for coronary heart disease in diabetic patients. *Journal of Endocrinological Investigation*. 2007;30:721-725

38. Bundo M, Munoz L, Perez C, Montero JJ, Montell N, Toran P, Pera G. Asymptomatic peripheral arterial disease in type 2 diabetes patients: A 10-year follow-up study of the utility of the ankle brachial index as a prognostic marker of cardiovascular disease. *Annals of Vascular Surgery*. 2010;24:985-993

39. Mlacak B, Blinc A, Pohar M, Stare J. Peripheral arterial disease and ankle-brachial pressure index as predictors of mortality in residents of Metlika County, Slovenia. *Croatian Medical Journal*. 2006;47:327-334

40. Ulmer H, Kelleher C, Diem G, Concin H. Long-term tracking of cardiovascular risk factors among men and women in a large population-based health system: the Vorarlberg Health Monitoring & Promotion Programme. *Eur Heart J*. 2003;24:1004-1013

41. Xu Y, Li J, Luo Y, Wu Y, Zheng L, Yu J, Ma J, Gu J, Hu D. The association between ankle-brachial index and cardiovascular or all-cause mortality in metabolic syndrome of elderly Chinese. *Hypertension Research - Clinical & Experimental*. 2007;30:613-619

42. Hasimu B, Li J, Nakayama T, Yu J, Yang J, Li X, Hu D. Ankle brachial index as a marker of atherosclerosis in Chinese patients with high cardiovascular risk. *Hypertens Res*. 2006;29:23-28

43. Li J, Luo Y, Xu Y, Yang J, Zheng L, Hasimu B, Jinming Y, Hu D. Risk factors of peripheral arterial disease and relationship between low ankle-brachial index and mortality from all-cause and cardiovascular disease in Chinese patients with type 2 diabetes. *Circulation Journal*. 2007;71:377-381

44. Yokoyama H, Sone H, Honjo J, Okizaki S, Yamada D, Shudo R, Shimizu H, Moriya T, Haneda M. Relationship between a low ankle brachial index and all-cause death and cardiovascular events in subjects with and without diabetes. *Journal of Atherosclerosis & Thrombosis*. 2014;21:574-581

45. Tanaka S, Uejima T, Sawada H. The predictive value of the borderline ankle-brachial index for long-term clinical outcomes: An observational cohort study. *Atherosclerosis*. 2016;250:01

46. Wang Y, Mou Q, Zhao D, Xu Y, Hu D, Ma H, Liu J, Guo X, Li J. Predictive value of ankle-brachial index and blood glucose on the outcomes of six-year all-cause mortality and cardiovascular mortality in a Chinese population of type 2 diabetes patients. *International Angiology*. 2012;31:586-594

47. Guo WW, Li J, Yu JM, Luo YY, Liu H, Zheng LQ, Buaijiaer H, Li XK, Hu DY. [The relationship of ankle brachial index to all-cause and cardiovascular disease mortality in Chinese male patients with hypertension]. *Chung-Hua Yu Fang i Hsueh Tsa Chih [Chinese Journal of Preventive Medicine]*. 2007;41:487-491

48. Newman AB, Shemanski L, Manolio TA, Cushman M, Mittelmark M, Polak JF, Powe NR, Siscovick D. Ankle-Arm Index as a Predictor of Cardiovascular Disease and Mortality in the Cardiovascular Health Study. *Arteriosclerosis, Thrombosis, and Vascular Biology*. 1999;19:538-545

49. Diederichsen AC, Rasmussen LM, Sogaard R, Lambrechtsen J, Steffensen FH, Frost L, Egstrup K, Urbonaviciene G, Busk M, Olsen MH, Mickley H, Hallas J, Lindholt JS. The Danish Cardiovascular Screening Trial (DANCAVAS): study protocol for a randomized controlled trial. *Trials [Electronic Resource]*. 2015;16:554

50. Kallio M, Forsblom C, Groop PH, Groop L, Lepantalo M. Development of new peripheral arterial occlusive disease in patients with type 2 diabetes during a mean follow-up of 11 years. *Diabetes Care*. 2003;26:1241-1245

51. Hyun S, Forbang NI, Allison MA, Denenberg JO, Criqui MH, Ix JH. Ankle-brachial index, toe-brachial index, and cardiovascular mortality in persons with and without diabetes mellitus. *Journal of Vascular Surgery*. 2014;60:390-395

52. Kusunose K, Sato M, Yamada H, Saijo Y, Bando M, Hirata Y, Nishio S, Hayashi S, Sata M. Prognostic Implications of Non-Invasive Vascular Function Tests in High-Risk Atherosclerosis Patients. *Circulation Journal*. 2016;80:1034-1040

53. Merino J, Clara A, Planas A, De Moner A, Gasol A, Contreras C. Influence of an increased ankle/brachial index on cardiovascular risk and survival in adult men with no peripheral arterial disease. [Spanish]. *Angiologia*. 2012;64:1-6

54. Feinkohl I, Keller M, Robertson CM, Morling JR, Williamson RM, Nee LD, McLachlan S, Sattar N, Welsh P, Reynolds RM, Russ TC, Deary IJ, Strachan MW, Price JF, Edinburgh Type 2 Diabetes Study I. Clinical and subclinical macrovascular disease as predictors of cognitive decline in older patients with type 2 diabetes: the Edinburgh Type 2 Diabetes Study. *Diabetes Care*. 2013;36:2779-2786

55. Ohman EM, Bhatt DL, Steg PG, Goto S, Hirsch AT, Liau C-S, Mas J-L, Richard A-J, Röther J, Wilson PWF. The REduction of Atherothrombosis for Continued Health (REACH) Registry: An international, prospective, observational investigation in subjects at risk for atherothrombotic events-study design. *American Heart Journal*. 2006;151:786.e781-786.e710

56. Velescu A, Clara A, Penafiel J, Ramos R, Marti R, Grau M, Degano IR, Marrugat J, Elosua R. Adding low ankle brachial index to classical risk factors improves the prediction of major cardiovascular events: The REGICOR study. *Atherosclerosis*. 2015;241:357-363

57. Alonso-Bouzon C, Carcaillon L, Garcia-Garcia FJ, Amor-Andres MS, El Assar M, Rodriguez-Manas L. Association between endothelial dysfunction and frailty: the Toledo Study for Healthy Aging. *Age*. 2014;36:495-505

58. Hyde Z, Mylankal KJ, Hankey GJ, Flicker L, Norman PE. Peripheral arterial disease increases the risk of subsequent hip fracture in older men: the Health in Men Study. *Osteoporosis International*. 2013;24:1683-1688

59. Bo M, Zanocchi M, Poli L, Molaschi M. The ankle-brachial index is not related to mortality in elderly subjects living in nursing homes. *Angiology*. 2005;56:693-697

60. Grøndal N, Søgaard R, Lindholt JS. Baseline prevalence of abdominal aortic aneurysm, peripheral arterial disease and hypertension in men aged 65–74 years from a population screening study (VIVA trial). *British Journal of Surgery*. 2015;102:902-906

## Table S1 – Search strategy

Master search strategy designed for OVID Medline, adapted for Embase via OVID and CINAHL via EBSCO.

| **No** | **Search term** |
| --- | --- |
| 1 | subclavian.ti,ab. |
| 2 | stenosis.ti,ab. |
| 3 | 1 and 2 |
| 4 | inter-arm.ti,ab. |
| 5 | interarm.ti,ab. |
| 6 | 4 or 5 |
| 7 | BP*.ti. |
| 8 | differen*.ti. |
| 9 | 7 and 8 |
| 10 | ABI.ti,ab. |
| 11 | ABPI.ti,ab. |
| 12 | ankle brachial pressure.ti,ab. |
| 13 | ankle brachial pressure index.ti,ab. |
| 14 | 10 or 11 or 12 or 13 |
| 15 | 3 or 6 or 9 or 14 |
| 16 | exp Cohort Studies/ |
| 17 | cohort.mp. |
| 18 | follow up.mp. |
| 19 | 16 or 17 or 18 |
| 20 | 15 and 19 |

## Table S2 – Summary of consensus ethnicity classifications for studies

|  | **Study** | **Analysis name** | **Country of Study Origin** | **Reported Ethnicities** | **Agreed Study classifications** |
| --- | --- | --- | --- | --- | --- |
| 1 | Mid Devon Hypertension cohort^1^ | Mid Devon | England | White British | WHITE |
| 2 | Mid Devon Hypertension cohort^2^ | Mid Devon HT | England | White British | WHITE |
| 3 | Chronic Disease  Detection and Management in the Elderly^3^ | Elderly Chinese | China | Chinese/East Asian | CHINESE |
| 4 | Vietnam Experience Study^4^ | VIETNAM | USA | White  Black  Other (Hispanics, Asians, Pacific Islanders, American Indians, and Alaskan Natives) | WHITE  AFRICAN-AMERICAN  OTHER |
| **5** | Diabetes Alliance for Research in England^5^ | DARE | England | White British 96% | WHITE  OTHER |
| **6** | Aspirin in Asymptomatic Atherosclerosis^6^ | AAA | Scotland | N/S*  2011 census reports: *“The African, Caribbean or Black groups made up 1% of the population of Scotland in 2011, Mixed or multiple ethnic groups represented 0.4% and other ethnic groups 0.3% of the total population.”* | WHITE |
| **7** | Invecchiare in Chianti^7^ | InCHIANTI | Italy | N/S: expert advised assume all white | WHITE |
| **8** | Heinz Nixdorf Recall Study^8^ | Heinz Nixdorf | Germany | N/S: author advised all Caucasian | WHITE |
| **9** | Second Manifestations of ARTerial disease study^9^ | SMART | The Netherlands | White 53%  Not recorded 44% | WHITE  OTHER |
| **10** | Lipid Research Clinics^10^ | LRC | USA | White 98% | WHITE  OTHER |
| **11** | San Diego Population Study^11^ | San Diego | USA | Caucasian  Hispanic  African American  Other | WHITE  HISPANIC  AFRICAN-AMERICAN  OTHER |
| **12** | Fuencarral Health Center^12^ | Fuencarral | Spain | N/S: *author advised vast majority were Caucasian* | WHITE |
| **13** | Peripheral Arterial Disease Study^13^ | ARTPER | Spain | Caucasian (98%)  Black  Arabic  Asian  Latin  Other | WHITE  OTHER |
| **14** | Epidemiology of dementia in Central Africa^14^ | EPIDEMCA | Central African Republic  Republic of the Congo | All ethnic Black African by tribe:  *Expert advised classify as Black African* | BLACK AFRICAN |
| **15** | Multi Ethnic Study of Atherosclerosis^15^ | MESA | USA | Non-Hispanic white  African American  Hispanic  Chinese American | WHITE  AFRICAN-AMERICAN  HISPANIC  OTHER |
| **16** | Lifestyle Interventions and Independence for Elders study^16^ | LIFE | USA | Non-Hispanic White  African American  Asian  Hispanic  Other | WHITE  AFRICAN-AMERICAN  HISPANIC  OTHER |
| **17** | Limburg PAOD Study^17^ | Limburg PAOD | The Netherlands | N/S: *author advised majority white Caucasian* | WHITE |
| **18** | Men born in 1914.^18^ | Men Born 1914 | Sweden | N/S: *author advised can assume all white European* | WHITE |
| **19** | Action for Health in Diabetes^19^ | Look AHEAD | USA | African-American 10%  White 69%  Hispanic 18%  Multiple/Other 2% | AFRICAN-AMERICAN  WHITE  HISPANIC  OTHER |
| **20** | Kinmen Health Survey^20^ | Kinmen | Kinmen (Republic of China) | N/S: *author confirmed all ethnic Chinese* | CHINESE |
| **21** | Chicago Walking and Leg Circulation Study^21^ | WALCS | USA | White 75%  African American 19%  Hispanic 2% | AFRICAN-AMERICAN  WHITE  HISPANIC  OTHER |
| **22** | Viborg Women Cohort^22^ | ViWoCo | Denmark | Caucasian  Asiatic (n=4)  Other (n=3) | WHITE  OTHER |
| **23** | Surrogate markers for Micro- and Macrovascular hard endpoints as Innovative diabetes tools^23^ | SUMMIT | England | N/S: author confirmed 99% White British | WHITE |
| **24** | Improving interMediAte RisK management study^24^ | MARK | Spain | N/S: author confirmed all European Caucasian | WHITE |

*N/S = Not specified

### Notes on classification of ethnicity

Since the classifications of ethnicity used for different cohorts varied, and could not directly be mapped to each other, we classified ethnicity for individual participants according to the following steps:

**First,** we identified the most prevalent ethnic groupings within the IPD dataset and adopted these as the basis for our classification (namely White, African American, Hispanic American, East Asian, Black African and other – the latter including any not fitting one of the preceding groups). Where individual categories of ethnicity were unclear from the data and supporting information supplied, we sought advice from study authors to confirm these.

**Second**, where ethnicity was confirmed to be unknown, we agreed a conservative approach and classified such cases as “other” ethnicity.

**Finally**, for a total of only 453 (0.8%) cases whose ethnicity did not correspond to our chosen classification groups, we elected for this study to also code them as “other” ethnicity.

## Table S3 – Quality assessment – modified QUIPS tool

| **Domains** | **Prompting items for Consideration** | **Ratings** |
| --- | --- | --- |
| **Study Participation** | **Summarising question:**  ***Are the participants in the sample representative of the population that they are drawn from, and intended to represent?***  Clues:   1. *Recruitment rate*: Is there adequate participation in the study by eligible persons? 2. *Description of population:* Is there adequate description of the source population or population of interest? 3. *Baseline demographics:* Is there a description or table describing the baseline study sample? 4. *Method of sampling:* Is there a description of how the population was sampled and/or how participants were recruited? 5. *Time to recruit:* Is there an adequate description of the time period and place of recruitment? 6. *Are exclusions excessive?* Is there an adequate description of inclusion and exclusion criteria? | **Unclear bias**: The relationship between the BP and/or IAD and outcome may be different for participants and eligible nonparticipants, but we cannot tell  **High bias:** The relationship between the BP and/or IAD and outcome is very likely to be different for participants and eligible nonparticipants  **Low bias**: The relationship between the BP and/or IAD and outcome is unlikely to be different for participants and eligible nonparticipants |

| **Study Attrition** | **Summarising question:**  ***Do the participants with follow up data represent the population enrolled in the study/cohort?***  Clues:   1. *Loss to follow up:* Is there an adequate response rate for study participants? How many are lost at follow up? 2. *Completeness of follow up:* Is there a description of attempts to collect information on participants who dropped out? 3. *Recording of losses to follow up:* Are reasons for loss to follow-up provided (in a flow chart or text)? Are those lost to follow up described? 4. *Differences between those completing and those lost to follow up:* Are there any important differences between participants who completed the study (were followed up) and those who did not? | **Unclear bias**: The relationship between the BP and/or IAD and outcome may be different for participants and eligible nonparticipants, but we cannot tell  **High bias:** The relationship between the BP and/or IAD and outcome is very likely to be different for participants and eligible nonparticipants.  **Low bias**: The relationship between the BP and/or IAD and outcome is unlikely to be different for participants and eligible non-participants |
| --- | --- | --- |

| **Prognostic Factor**  **Measurement (i.e. blood pressure measurement)**  *Uncertainties identified here should be flagged to the data cleaning team and if needed referred to study authors for clarification* | **Summarising question:**  ***Has the method of blood pressure measurement been adequately described and is it the same for all participants?***  Clues:   1. *Definition:* Is there a clear definition or description of the method of BP measurement? 2. *Validity:* Does the method of BP measurement appear valid and reliable? 3. *Uniformity:* Is the method and setting for measurement of BP the same for all study participants? 4. *Completeness of baseline data:* Did an adequate proportion of the study sample have complete BP data? | **Unclear bias**: The measurement of the BP may be different for different levels of the outcome of interest, but we cannot tell  **High bias**: The measurement of the BP is very likely to be different for different levels of the outcome of interest  **Low bias**: The measurement of the BP is unlikely to be different for different levels of the outcome of interest |
| --- | --- | --- |
| **Outcome Measurement**  *(survival: all cause and cardiovascular deaths, cardiovascular non-fatal events, strokes, changes in cognitive measures)* | **Summarising question:**  ***Are the methods of determining outcomes clearly described, and applied similarly to all participants?***  Clues:   1. *Definition:* Is a clear definition of the outcomes provided? 2. *Validity:* Is the method(s) of outcome measurement used valid and reliable? 3. *Uniformity:* Is the method of outcome measurement the same for all study participants? | **Unclear bias**: The measurement of the outcome may be different related to the baseline level of the BP, but we cannot tell  **High bias**: The measurement of the outcome is very likely to be different related to the baseline level of BP  **Low bias**: The measurement of the outcome is unlikely to be different related to the baseline level of the BP |

**BP** = blood pressure, **IAD** = inter-arm blood pressure difference.

## Derivation of the modified QUIPS tool

We undertook an evaluation session with PPI representatives to explore the applicability of the QUIPS tool to a randomly chosen cohort study. This highlighted the redundancy of some domains of assessment with respect to IPD analysis, namely statistical treatment and reporting of results, since we were undertaking our own analyses within this study. Note was also taken of comments designed to improve the legibility of the tool. The modified tool was presented to two co-applicants’ meetings and adapted following each meeting with advice and approval from the IMG. Key changes were:

1. Restriction of the QUIPS tool to four agreed key risk of bias items:

- study participation
- study attrition
- method of blood pressure measurement (prognostic factor measurement)
- outcome measurement (methods of adjudication of fatal and non-fatal events)

1. Addition of a summarising question followed by clues to guide assessment in each domain.
2. Replacement of High/moderate/low categories of assessment with unclear/high/low.
3. Provision for adjudication of differences by a third author was made (but not required).

**Note:** CEC did not quality assess studies that he had authored to minimise bias.

## Table S4 – Characteristics of included studies

| **Study name** | **Period of patient recruitment**  **/Duration of trial** | **Sample size** | **Country of origin** | **Eligibility criteria** | **Primary outcome measure** | **Blood pressure measurement method for IAD** | **Intended maximum duration of follow up** | **Definition of hypertension** | **Definition of diabetes** | **Definition of cardiovascular death and non-fatal cardiovascular event** |
| --- | --- | --- | --- | --- | --- | --- | --- | --- | --- | --- |
| Aspirin in Asymptomatic Atherosclerosis (AAA)^6^ | April 1998-October 2008 | 3350 | Scotland | Males and females, aged 50-75 years, living in central Scotland, free of clinical cardiovascular disease with an ABI < 0.95 | Initial fatal or non-fatal coronary event or stroke or revascularisation | Single pair of sequential BP readings recorded using a Doppler probe (Huntleigh Healthcare, Cardiff) and aneroid desk sphygmomanometer (Accoson;A.C. Cossor Ltd, London, UK) with patient supine | 5 years with extended follow-up of 4.5 years.  Mean 8.2 years | N/S | Self-reported diabetes | *Cardiovascular death*: Definite or probable fatal MI, death due to IHD, or fatal stroke due to infarction.  *Non-fatal events*:  MI, stroke or TIA, coronary or peripheral revascularisation. angina, PAD |
| Peripheral Arterial Disease Study (ARTPER)^13^ | October 2006-ongoing (at time of publication) | 3786 | Spain | Males and females, aged > 49 years, registered at 28 Primary Health Care centres in Barcelona. | Incidence of cardiovascular events and death | Two pairs of sequential BP readings recorded with sphygmomanometer (Welch Allyn, model Ds-66); results rounded to nearest 2 mmHg) with patient supine | Mean follow-up was 4 years | N/S | N/S | *Cardiovascular death:*  Not defined.  *Non-fatal events*:  Coronary artery disease: acute MI or angina, stroke or TIA, symptomatic aneurysm of abdominal aorta, vascular surgery, or cardiovascular morbidity |
| Chicago Walking and Leg Circulation Study (WALCS)^21^ | 1998-2000 | 740 | USA | Patients without lower extremity peripheral artery disease who were recruited for the non-PAD comparator group. | Subclavian stenosis as a marker for total and cardiovascular disease mortality | Two pairs of sequential BP readings recorded using a 12-cm pneumatic cuff and a handheld Doppler probe (Nicolet Vascular Pocket Dop II, Golden, Colo) with patient supine | Mean follow-up was 4.8 years. | Patient history or use of BP lowering therapy | Patient history or use of oral antidiabetic drugs and/or insulin | *Cardiovascular death*:  Any fatal cardiovascular cause.  *Non-fatal events*:  MI, stroke, TIA, coronary or peripheral revascularisation, congestive heart failure, PAD, angina |
| Diabetes Alliance for Research in England (DARE)^5^ | October 30th 2007-February 12th 2010 | Type 1 or 2 diabetes: 727; Non-diabetic controls: 285 | England | Males and females, with type 1 or 2 diabetes and non-diabetic controls, living in Devon | Inter-arm difference in BP and association with target organ disease and mortality | Four pairs of simultaneous BP readings recorded using a pair of automated sphygmomanometers (Omron 705IT; Omron Matsusaka, Japan) swapped after two readings, with patient seated | 5 years. Median follow-up: 52 months | Use of BP lowering medication or recruitment  SBP ≥ 140 mmHg or DBP ≥80 mmHg | Diagnosis of diabetes recorded in primary or secondary care diabetes registers | *Cardiovascular deaths*:  MI, cardiac failure or ischemic stroke.  *Non-fatal events:*  PAD, cardiovascular and cerebrovascular events, amputation |
| Chronic Disease  Detection and Management in the Elderly (Elderly Chinese)^3^ | 2006-2008 | 3133 | China | Males and females, aged ≥ 60 years, living in a newly urbanized suburban town 30km from Shanghai | Predictive value of BP for cardiovascular morbidity and mortality | Two pairs (first pair discarded) of simultaneous BP readings recorded using a Vascular Profiler-1000 device (Omron, Kyoto, Japan) with patient supine | Followed up for vital status and cause of death until June 2011. Median follow-up: 4 years | SBP ≥140 mmHg,  DBP ≥90 mmHg (average of 3 readings) or use of BP lowering drugs | Plasma glucose ≥7.0 mmol/L fasting or  11.1 mmol/L non-fasting, or use of antidiabetic agents | *Cardiovascular deaths:*  Stroke, MI or other cardiovascular diseases, cerebrovascular disease and PAD |
| Epidemiology of dementia in Central Africa (EPIDEMCA)^14^ | November 2011- December 2012 | 2002 | Central African Republic/ Republic of Congo | Males and females, aged ≥ 65 years living in areas of Central African Republic and Republic of Congo | Diagnosis of dementia and Alzheimer's disease and associated risk factors | Two pairs of BP measurements recorded in each arm using standard mercury sphygmomanometer, as part of ABI protocol, with patients supine. BP rounded to nearest 5 mmHg | 2-3 years | Self-reported BP lowering treatment;  SBP ≥140 mmHg or DBP ≥90 mmHg | Self-reported or blood glucose >126 mg/dL fasting or >200 mg/dL in non-fasting | *Cardiovascular death:*  Stroke, MI or other cardiovascular or cerebrovascular diseases – based on interview of relatives during verbal autopsy at follow-up.  Non-fatal events: Stroke, MI, other heart disease |
| Fuencarral Health Center^12^ | 2003-2004 | 1361 | Spain | Males and females, aged 60-79 years, with no known PAD | Low ABI and incidence of death due to cardiovascular causes | BP measured Doppler 8-MHz probe (Hadeco, Kawasaki, Japan) and calibrated mercury sphygmomanometer as part of ABI protocol with patient supine | Mean follow-up 49.8 months | SBP ≥140 mmHg, DBP ≥90 mmHg or use of BP lowering treatment | Baseline glucose ≥126 mg/dl (>7 mmol/L) on 2 occasions or use of antidiabetic agents | *Cardiovascular death:*  Fatal stroke, MI, sudden death without other cause, death after vascular surgery or procedure, death attributed to heart failure, bowel or limb infarction, any other death not categorically attributed to a non-vascular cause  *Non-fatal events*:  MI, stroke or cardiovascular event |
| Heinz Nixdorf Recall Study^8^ | 2000-2003 | 4735 | Germany | Males and females, aged 45-74 years, in an unselected urban population from the Ruhr area | Coronary artery calcium as predictor for fatal and non-fatal MI.  Secondary endpoints included ABI as a stroke predictor factors | BP measured sequentially using Doppler probe (Logidop, Kranzbuhler, Germany) with patients supine | Mean follow up: 109 months | SBP >140mmHg or DBP >90mmHg | Existing diagnosis or use of anti-diabetic medication | *Cardiovascular death or non-fatal event:*  First occurrence of MI based on symptoms, ECG signs, and enzymes, supported by necropsy if fatal |
| Invecchiare in Chianti (InCHIANTI)^7^ | August 1998-March 2000 | 1270 | Italy | Males and females, aged ≥ 65 years, living in Greve and Bagno | Physiological factors influencing walking ability | Single pair of sequential BP readings using standard mercury sphygmomanometer, with patients supine. BP rounded to nearest 5 mmHg | N/S | Self-reported, existing, recorded diagnosis or use of BP lowering medication or SBP ≥140 mmHg or DBP ≥90 mmHg | Self-reported, existing recorded diagnosis, or use of anti-diabetic medication, or fasting glucose >7.0 mmol/L | *Cardiovascular death:*  Not defined.  *Non-fatal events:*  Diagnosis of heart disease, MI or angina, stroke or TIA |
| Kinmen Health Survey^20^ | 2002-2012 | 1329 | Kinmen (Republic of China) | Community living individuals, aged ≥ 40 years | Association between ABI and brachial-ankle pulse and mortality | Simultaneous BP readings were recorded. Device or measurement position not stated | Median follow up: 10 years | N/S | N/S | *Cardiovascular death*:  “Death by cardiovascular cause”  *Non-fatal events:*  Not described |
| Lifestyle Interventions and Independence for Elders (LIFE) study^16^ | 2010-2011/  2.6 years | 1635 | USA | Ambulant community dwelling individuals, aged 70-89 years with a sedentary lifestyle (<20min per week physical activity) | Major mobility disability  Secondary: Association between ABI and cognitive function | Two pairs of sequential BP measurements recorded in each arm using handheld Doppler, with patients supine | 2 years | Self-reported or measurement | Self-reported | *Cardiovascular fatal or*  *non-fatal events:*  MI, angina, stroke or TIA, carotid artery disease, congestive heart failure or PAD requiring hospitalisation, outpatient revascularisation for PAD, ruptured abdominal aortic aneurysm |
| Limburg PAOD Study^17^ | N/S | 3649 | The Netherlands | Males and females, from 18 General Practice clinics, aged 40-75 years, in Limburg | Progressive limb ischaemia, non-fatal cardiovascular morbidity and mortality | BP measured in both arms using pocket Doppler device (Huntleigh Mini Dopplex D500, 8Mhxz) and a sphygmomanometer | Mean follow up: 7.2 years | Existing recorded diagnosis | Existing recorded diagnosis | *Cardiovascular deaths:*  MI, sudden deaths, strokes, aortic aneurysms and death due to PAD complications  *Non-fatal events:*  MI, angina, stroke, aortic aneurysm., PAD, vascular surgery or intervention, amputations due to PAD |
| Lipid Research Clinics (LRC)^10^ | 1978-1979 | 624 | USA | Community dwelling individuals participating in the Lipid Research Clinics study, Southern California | Subclavian stenosis as a marker for total and cardiovascular disease mortality | Two pairs of sequential BP measurements were recorded, twice, using a mercury-in-Silastic gauge | 24.9 years. Mean follow up 9.8 years | Patient history or use of BP lowering therapy | Patient history or use of oral antidiabetic drugs and/or insulin | *Cardiovascular deaths*:  Death by any cardiovascular cause  *Non-fatal events*:  MI, stroke, TIA, or revascularization of the coronary, carotid, or lower-extremity arteries |
| Improving interMediAte RisK management (MARK) study^24^ | N/S | 2688 | Spain | Males and females living in 3 regions of Spain, aged 35-74 years. Free of atherosclerotic disease, with an intermediate cardiovascular risk (10-year coronary risk of 5-15% or vascular death risk of 3-5%) selected at random | Incidence of vascular events | Three pairs of BP measurements in each arm, using an OMRON 705, with patients seated | 10 years | Patient reported, or use of BP lowering medications or SBP ≥140mmHg or DBP ≥90mmHg | Patient reported, or use of antidiabetic treatment or fasting glucose ≥ 126 mg/dL | *Cardiovascular death:*  not defined  *Non-fatal events*:  Stroke or TIA, MI, angina, or revascularisation procedure |
| Action for Health in Diabetes (Look AHEAD)^19^ | June 2001-March 2004 | 479 | USA | Overweight and obese individuals with type 2 diabetes aged 45-76 years, and had a body mass index, 25 kg/m2, or ≥27 kg/m2 if taking insulin | A composite cardiovascular outcome: cardiovascular death, non-fatal MI, non-fatal stroke, hospitalized angina  Secondary: Cognitive function | Two pairs of sequential BP measurements recorded in each arm, using continuous wave Doppler with a standard mercury sphygmomanometer, with patients supine | 4-5 year follow up | SBP ≥140 mmHg, ≥DBP > 90 mmHg or taking BP lowering medication | Self-reported verified from medical records, current treatment, or fasting glucose of ≥126 mg/dL | *Cardiovascular death:*  MI, congestive heart failure, death after cardiovascular intervention, surgery or due to arrhythmia, stroke, presumed cardiovascular death, rapid unexplained cardiovascular death.  *Non-fatal events:*  Stroke, MI, angina, coronary artery bypass grafting or percutaneous coronary intervention, congestive heart failure, carotid endarterectomy, peripheral arterial bypass or angioplasty |
| Men born in 1914.^18^ | 1982-1983 | 474 | Sweden | Males, born in even months in 1914 and residing in Malmö in 1982–1983 | Prevalence of PAD and cardiovascular mortality | Two BP measurements, recorded using mercury-in-silastic strain gauges, with patients supine | 14.3 years. Median: 13.2 years | SBP ≥160mmHg, DBP ≥100mmHg or use of BP lowering medications | History of diabetes or fasting blood glucose ≥ 6.1 mmol/L | *Cardiovascular death:*  MI or death from ischemic heart disease  *Non-fatal events:*  MI |
| Multi Ethnic Study of Atherosclerosis (MESA)^15^ | 2000-2002 | 6743 | USA | Males and females, aged 45-84 years, free of clinical cardiovascular diagnoses at baseline | Association of subclavian stenosis with markers of cardiovascular disease | Single pair of sequential BP measurements, using hand-held Doppler instrument and 5-mHz probe, with patients supine | N/S | Self-reported history with use of BP lowering medications, or SBP ≥140mmHg or DBP ≥ 90mmHg | Fasting blood glucose ≥126 mg/dl or use of oral hypoglycemic agents or insulin | *Cardiovascular death*:  Death due to atherosclerotic coronary heart disease, stroke, other cardiovascular disease.  *Non-fatal* events:  Stroke, TIA, MI, angina, revascularisation procedure |
| Mid Devon Hypertension cohort^1^ | 9 November 1999 to 17 June 2002 | 230 | England | Males and females, receiving treatment for hypertension in rural general practices in Devon | Cardiovascular events and deaths from all causes | Single pair of sequential BP measurements, using standard calibrated mercury sphygmomanometer, with patients seated | 11.4 years. Median follow up: 9.8 years | SBP ≥160 mmHg or DBP ≥100 mmHg or ≥140/90 mmHg with target organ damage, diabetes or coronary heart disease risk score > 15% | N/S | *Cardiovascular death*.  Not defined  *Non-fatal events*:  Stroke, TIA, MI or angina |
| Mid Devon Pilot study^2^ | May 1994-Ocober 1995 | 280 | England | Males and females, aged 19-88 years (median age: 60 years), attending a general practice for re-registration medicals | Inter-arm difference, new diagnosis of angina, MI, cerebrovascular event or death | Single pair of sequential BP measurements, using standard calibrated mercury sphygmomanometer, with patients seated | 5.6 years | N/S | N/S | *Cardiovascular fatal and non-fatal events:*  Cardiovascular disease-related causes. Ischaemic heart disease events and cerebrovascular events |
| San Diego Population Study^11^ | 1994-1998 | 2404 | USA | Males and females, aged 29-91 years, attending a clinic for assessment of PAD and venous disease | Prevalence of PAD | Two pairs of BP measurements, using a continuous-wave Doppler ultrasound, with patients supine | N/S | SBP ≥140 mmHg or DBP ≥ 90 mmHg or use of BP lowering medications | Self-reported or use of antidiabetic medications | *Cardiovascular death:*  not defined  *Non-fatal events:*  MI, stroke, angina, coronary angioplasty or bypass graft, or carotid endarterectomy |
| Second Manifestations of ARTerial disease (SMART) study^9^ | January 2002 – February 2014 | 7344 | The Netherlands | Males and females, aged 18-80 years, referred to University Medical Center Utrecht, for treatment of clinically manifest vascular disease or cardiovascular risk factors | 3-point MACE (combination of non-fatal myocardial infarction, non-fatal stroke and death from vascular disease), total mortality and vascular mortality | Single pair of sequential BP measurements, using a Vasoguard Doppler probe, with patients supine | Mean follow-up:5.9 years | Blood pressure >140/90 mmHg at baseline or the use of blood pressure lowering medication. | Recorded or self-reported diagnosis, or blood glucose lowering drugs, or fasting glucose >7 mmol/L at recruitment *plus* initiation of glucose lowering drugs within first year of follow-up.  Type 1 diabetes excluded. | *Cardiovascular death:*  Death from stroke, MI, congestive heart failure, rupture of abdominal aortic aneurysm or vascular death from other causes  *Non-fatal events:*  Stroke (infarction or haemorrhagic), MI, retinal infarction, heart failure  (see published data supplement for full definitions)^9^ |
| Surrogate markers for Micro- and Macrovascular hard endpoints as Innovative diabetes tools (SUMMIT)^23^ | November 2010 – June 2013 | 596 | England | Adults over 18 with and without diabetes and/or cardiovascular disease |  | 6 pairs of simultaneous BP readings using two Omron 705 devices swapped after 3 readings, with patients supine | N/S | Self-reported history of hypertension | HbA1c ≥ 48 mmol/mol | *Cardiovascular death:*  Fatal MI |
| Viborg Women Cohort (ViWoCo)^22^ | October 2011-January 2013 | 1474 | Denmark | Females born in 1936, 1941, 1946 and 1951 living in the Municipal of Viborg, Denmark | Presence of cardiovascular disease and diabetes mellitus | One pair of simultaneous BP readings, using Omron M2 devices, with patients supine, rounded to nearest 2mmHg | Median follow-up 3.3 years | SBP ≥160 mmHg or DBP ≥100 mmHg | HbA1c ≥ 48 mmol/mol | *Cardiovascular death:*  Fatal event as below  *Non-fatal event:*  MI or ischaemic stroke leading to hospitalisation |
| Vietnam Experience Study^4^ | 1986 | 4419 | USA | Male US army veterans who participated in the Vietnam war | Inter-arm differences, all-cause and cardiovascular mortality | Two pairs of sequential BP measurements, using standard mercury sphygmomanometer, with patients seated | 15 years | SBP ≥140 mmHg, DBP ≥90 mmHg or use of BP lowering medication | Fasting plasma glucose ≥ 7.0 mmol/l and/or use of medication for diabetes | *Cardiovascular death:*  Death due to major cardiovascular disease. |
| ABI = ankle-brachial index, BP = BP, DBP = diastolic BP, IHD = ischaemic heart disease, MI = myocardial infarction, N/S = not stated, PAD = peripheral arterial disease, SBP = systolic BP  TIA = transient ischaemic attack, ECG = electrocardiogram | | | | | | | | | | |

## Table S5 – Potentially eligible cohorts excluded with reasons

|  | **Study** | **Notes** |
| --- | --- | --- |
| ***INVITED AND AGREED – DATA NOT RECEIVED BY DEADLINE*** | | |
| **25** | Baltimore Longitudinal Study of Aging (BLSA)^25^ |  |
|  |  |  |
| ***ELIGIBLE AND RESPONDED BUT DID NOT SUPPLY DATA*** | | |
| **26** | German epidemiological trial on Ankle Brachial Index (getABI)^26^ |  |
| **27** | HOORN study^27^ |  |
| **28** | Mexican Teachers' Cohort^28^ |  |
|  |  |  |
| ***ELIGIBLE BUT NOT OBTAINED*** | | |
| **29** | Framingham^29^ | INTERPRESS could not meet required data transfer fees |
| **30** | Hisayama study^30^ | Author could not provide IPD, offered aggregate data only |
| **31** | INVADE^31^ | Author advised data no longer available |
| **32** | Rancho Bernardo^32^ | INTERPRESS could not meet required data transfer fees |
| **33** | Tosa Longitudinal Aging Study^33^ | Confirmed both arms measured. Prior agreement with local government collaborator did not permit data sharing |
| **34** | McKenna^34^ | 1990 study: data no longer available |

| ***ELIGIBLE BUT NEVER RESPONDED*** | | |
| --- | --- | --- |
| **35** | Honolulu 2000^35^ |  |
|  | | |
| ***ELIGIBILITY NEVER ESTABLISHED - NEVER RESPONDED TO ENQUIRIES WITH TWO FOLLOW-UP REMINDERS*** | | |
| **36** | Albacete^36^ | Unclear if both arms |
| **37** | Aosta study^37^ | Unclear if bilateral BP in database |
| **38** | Catalonia diabetes study^38^ | Both arm BPs measured, unclear in database |
| **39** | Mlacak^39^ | Methods unclear |
| **40** | Vorarlberg Health Monitoring and Promotion Program (VHM&PP)^40^ | Appears to have measured both arms, unclear if any outcome data |
| **41** | Xu^41^ | Methods unclear |
| **42** | Chinese ABI cohort^42^ | Both arms measured in methods paper, unclear if in database |
| **43** | Li et al^43^ | Both arms measured, unclear if in database |
| **44** | Yokoyama et al^44^ | No contact address found |
| **45** | Shinken database^45^ | Both arms measured, unclear if in database |
| **46** | Chinese diabetes study^46^ | No contact address found |
| **47** | Unamed Chinse cohort^47^ | No contact address found |
|  |  |  |

| ***COHORTS EXCLUDED AFTER DISCUSSION WITH AUTHORS*** | | |
| --- | --- | --- |
| **48** | Cardiovascular Health Study^48^ | No bilateral arm data |
| **49** | DANCAVAS^49^ | No outcome data |
| **50** | Helsinki Diabetes registry^50^ | Bilateral data only in original records, not study database |
| **51** | Hyun et al^51^ | Cohort selected from vascular labs |
| **52** | Kusunose et al^52^ | No bilateral arm data in database |
| **53** | Merino et al^53^ | Only ABI recorded in dataset, no bilateral arm data |
| **54** | The Edinburgh Type 2 Diabetes Study^54^ | Both arms measured – only one arm in database |
| **55** | REACH registry^55^ | No bilateral BP data in registry |
| **56** | REGICOR study^56^ | Only higher arm BP in database |
| **57** | Toledo Study for Healthy Aging^57^ | Only ABI not bilateral arm data |
| **58** | Health in Men study^58^ | Only one am in database |
| **59** | Nursing home^59^ | Both arms recorded but cannot access data anymore. |
| **60** | VIVA^60^ | No outcome data |

## Table S6 - Descriptive pooled baseline data from all studies

|  | **All participants** | | **sIAD <10mmHg** | | **sIAD ≥10mmHg** | | **OR (95%CI) for**  **sIAD ≥ 10 vs <10 mmHg** | ***P-*value** |
| --- | --- | --- | --- | --- | --- | --- | --- | --- |
|  | **N** | **Mean (SD)** | **N** | **Mean – (SD)** | **N** | **Mean – (SD)** |  |  |
| **Age (years)** | 53,827 | 60.3 (12.5) | 38,656 | 60.3 (12.5) | 15,171 | 60.5 (12.3) | 1.011 (1.009 to 1.013) | <0.001 |
| **Systolic blood pressure (mmHg)** | 53,826 | 138.3 (21.8) | 38,655 | 135.8 (20.8) | 15,171 | 144.8 (23.0) | 1.019 (1.018 to 1.020) | <0.001 |
| **Diastolic blood pressure (mmHg)** | 43,568 | 80.9 (11.8) | 31,131 | 80.0 (11.5) | 12,437 | 83.3 (12.3) | 1.016 (1.014 to 1.018) | <0.001 |
| **Body mass index (Kg/m^2^)** | 50,224 | 27.4 (5.1) | 36,420 | 27.1 (5.0) | 13,804 | 28.0 (5.2) | 1.033 (1.028 to 1.037) | <0.001 |
| **Total cholesterol (mmol/l)** | 49,412 | 5.4 (1.2) | 35,793 | 5.4 (1.2) | 13,619 | 5.4 (1.2) | 1.019 (1.000 to 1.038) | 0.047 |
| **HDL cholesterol (mmol/l)** | 39,882 | 1.4 (0.4) | 28,581 | 1.4 (0.4) | 11,301 | 1.3 (0.4) | 0.938 (0.886 to 0.994) | 0.032 |
|  | | | | | | | | |
|  |  | **N (%)** |  | **N (%)** |  | **N (%)** |  |  |
| **Female** | 53,827 | 25,724 (47.8) | 38,656 | 18,678 (48.3) | 15,171 | 7,046 (46.4) | 0.990 (0.948 to 1.033) | 0.636 |
| **Smoker** | 53,693 | 12,699 (23.7) | 38,567 | 8,842 (22.9) | 15,126 | 3,857 (25.5) | 1.014 (0.968 to 1.063) | 0.552 |
| **Hypertension** | 53,731 | 29,800 (55.5) | 38,572 | 20,605 (53.4) | 15,159 | 9,195 (60.7) | 1.301 (1.247 to 1.359) | <0.001 |
| **Diabetes** | 53,720 | 8,046 (15.0) | 38,570 | 5,607 (14.5) | 15,150 | 2,439 (16.1) | 1.150 (1.086 to 1.219) | <0.001 |
| **Cardiovascular disease** | 51,847 | 9,468 (18.3) | 36,986 | 6,401 (17.3) | 14,861 | 3,067 (20.6) | 1.060 (0.994 to 1.130) | 0.076 |
|  |  |  |  |  |  |  |  |  |
| **Ethnicity:** | 51,529 |  | 37,123 |  | 14,406 |  |  |  |
| **White (reference)** |  | 39,163 (76.0) |  | 26,915 (72.5) |  | 12,248 (85.0) |  | Global *P-*value  <0.001 |
| **African American** |  | 30,98 (6.0) |  | 2,402 (6.5) |  | 696 (4.8) | 1.195 (1.078 to 1.324) |  |
| **Hispanic American** |  | 1,956 (3.8) |  | 1,746 (4.7) |  | 210 (1.5) | 0.631 (0.537 to 0.742) |  |
| **Black African** |  | 1,008 (2.0) |  | 383 (1.0) |  | 625 (4.3) | 4.917 (1.070 to 22.581) |  |
| **East Asian** |  | 4,450 (8.6) |  | 4,141 (11.2) |  | 309 (2.1) | 0.235 (0.078 to 0.710) |  |
| **Other** |  | 1,854 (3.6) |  | 1,536 (4.1) |  | 318 (2.2) | 0.754 (0.662 to 0.860) |  |
| Cardiovascular disease includes ischaemic heart disease, cerebrovascular disease and peripheral arterial disease, SD = standard deviation,  sIAD = systolic inter-arm difference, OR = odds ratio derived from multi-level logistic regression taking account of study | | | | | | | | |

## Table S7 - Study level outcomes and attrition, all participants

| **Study** | **Total participants** | **Number of participants with all-cause mortality and time to death; n (%)** | **Number of participants with CVS cause mortality and time to death; n (%)** | **Number of participants who had at least one cardiovascular event and time to event; n (%)** | **Duration of follow-up to death or censorship (years); mean (sd), n; median [min, 25^th^ centile, 75^th^ centile, max]** | **Number of participants with missing mortality status and/or time to mortality; n (%)** | **Number of participants with missing CVS cause mortality status and/or time to mortality; n (%)** | **Number of participants with missing CVS event status and/or time to CVS event; n (%)** |
| --- | --- | --- | --- | --- | --- | --- | --- | --- |
| **AAA** | 3350 | 362 (10.8) | 102 (3.0) | 328 (9.8) | 8.22 (1.59), 3350; 8.38 [0.10, 7.54, 9.34, 10.45] | 0 (0.0) | 0 (0.0) | 0 (0.0) |
| **ARTPER** | 3748 | 286 (7.6) | 65 (1.7) | 243 (6.5) | 4.64 (0.99), 3748; 4.85 [0.14, 4.23, 5.20, 8.68] | 0 (0.0) | 0 (0.0) | 2 (0.1) |
| **Kinmen Health Survey** | 1329 | 115 (8.7) | 26 (2.0) | NR | 10.07 (1.31), 1329; 10.40 [0.44, 10.40, 10.40, 10.40] | 0 (0.0) | 0 (0.0) | NR |
| **Mid Devon Pilot study** | 83 | NR | NR | 21 (25.3) | 5.30 (1.98), 82; 6.20 [0.17, 4.78, 6.71, 7.02]^1^ | NR | NR | 1 (1.2) |
| **Mid Devon Hypertension cohort** | 247 | 65 (26.3) | 33 (13.4) | 67 (27.1) | 7.92 (3.41), 247; 9.73 [0.20, 5.20, 10.49, 11.46] | 0 (0.0) | 0 (0.0) | 0 (0.0) |
| **ViWoCo** | 1440 | 32 (2.2) | 5 (0.3) | 33 (2.3) | 3.44 (0.53), 1440; 3.30 [0.41, 2.96, 3.95, 4.22] | 0 (0.0) | 0 (0.0) | 0 (0.0) |
| **DARE** | 991 | 50 (5.0) | 16 (1.6) | NR | 4.33 (0.64), 991; 4.25 [0.11, 4.01, 4.64, 6.04] | 0 (0.0) | 0 (0.0) | NR |
| **EPIDEMCA** | 1029 | 110 (10.7) | 44 (4.3) | NR | 2.01 (0.37), 946; 2.05 [0.11, 1.98, 2.09, 2.69] | 83 (8.1) | 83 (8.1) | NR |
| **Heinz Nixdorf** | 4814 | 614 (12.8) | 169 (3.5) | 588 (12.2) | 10.68 (2.36), 4814; 11.48 [0.05, 10.16, 12.30, 14.24] | 0 (0.0) | 46 (1.0) | 0 (0.0) |
| **InCHIANTI** | 1453 | 402 (27.7) | 184 (12.7) | NR | 7.97 (2.38), 1317; 9.11 [0.10, 7.42, 9.31, 11.42] | 136 (9.4) | 136 (9.4) | NR |
| **Fuencarral Health Center** | 1361 | 72 (5.3) | 13 (1.0) | 61 (4.5) | 4.26 (0.80), 1301; 4.39 [0.46, 3.78, 4.80, 6.23] | 60 (4.4) | 61 (4.5) | 65 (4.8) |
| **LIFE** | 1635 | 133 (8.1) | 48 (2.9) | 161 (9.8) | 3.37 (0.87), 1635; 3.48 [0.00, 3.07, 3.98, 4.52] | 0 (0.0) | 0 (0.0) | 0 (0.0) |
| **Limburg** | 3035 | 468 (15.4) | NR | 538 (17.7) | 6.79 (1.48), 3034; 7.06 [0.00, 6.57, 7.56, 9.72] | 1 (0.0) | NR | 6 (0.2) |
| **Look AHEAD** | 342 | 30 (8.8) | 8 (2.3) | 54 (15.8) | 10.36 (1.69), 342; 10.90 [1.18, 10.76, 10.94, 11.05] | 0 (0.0) | 0 (0.0) | 0 (0.0) |
| **LRC** | 624 | 381 (61.1) | 74 (11.9) | NR | 16.38 (6.69), 624; 18.20 [0.11, 11.33, 22.48, 24. 89] | 0 (0.0) | 0 (0.0) | NR |
| **MARK** | 2495 | 26 (1.0) | 3 (0.1) | 96 (3.8) | 3.10 (0.34), 2471; 3.03 [0.07, 3.00, 3.14, 5.22] | 24 (1.0) | 24 (1.0) | 24 (1.0) |
| **MESA** | 6814 | 1161 (17.0) | 278 (4.1) | 911 (13.4) | 12.47 (2.50), 6809; 13.20 [0.17, 12.66, 13.74, 14.46] | 5 (0.1) | 5 (0.1) | 5 (0.1) |
| **Men born in 1914** | 480 | 479 (99.8) | 249 (51.9) | 168 (35.0) | 13.68 (7.56), 480; 13.70 [0.26, 7.69, 19.54, 30.40] | 0 (0.0) | 0 (0.0) | 0 (0.0) |
| **San Diego** | 2404 | 473 (19.7) | NR | NR | 17.58 (4.36), 2404; 19.21 [0.12, 18.27, 19.91, 20.89] | 0 (0.0) | NR | NR |
| **Elderly Chinese** | 3121 | 203 (6.5) | 93 (3.0) | NR | 4.08 (0.74), 3121; 3.95 [2.87, 3.81, 4.85, 5.02] | 0 (0.0) | 0 (0.0) | NR |
| **SMART** | 11,139 | 1766 (15.9) | 882 (7.9) | 1888 (16.9) | 7.84 (4.67), 11,139; 7.60 [0.00, 3.96, 11.31, 18.49] | 0 (0.0) | 0 (0) | 0 (0.0) |
| **SUMMIT** | 596 | 14 (2.3) | 1 (0.2) | 35 (5.9) | 2.93 (0.34), 596; 2.98 [0.57, 2.74, 3.14, 4.08] | 0 (0.0) | 0 (0.0) | 44 (7.4) |
| **VIETNAM** | 4462 | 250 (5.6) | 56 (1.3) | NR | 13.13 (2.59), 1555; 13.91 [0.23, 13.54, 14.25, 15.35] | 2907 (65.2) | 2907 (65.2) | NR |
| **WALCS** | 442 | 45 (10.2) | 12 (2.7) | NR | 3.98 (1.10), 431; 4.08 [0.16, 4.00, 4.42, 9.74] | 11 (2.5) | 11 (2.5) | NR |
| **Total** | 57,434 | 7537/57,351^2^ (13.1) | 2361/51,912^3^ (4.5) | 5192/41,579^4^ (12.5) | 8.21 (4.84), 54,124^5^; 7.50 [0.00, 4.00, 12.22, 30.40] | 3227/57,351^2^ (5.6) | 3965/51,912^3^ (7.6) | 147/41,579^4^ (0.4) |

This table presents descriptive data for all reported events, and all durations of follow-up, within the dataset, before censorship at 10-year follow-up

NR: Not reported. ^1^Time to first cardiovascular event or censorship (time to mortality not reported). ^2^Total participants for studies where all-cause mortality reported. ^3^Total participants for studies where cardiovascular mortality reported. ^4^Total participants for studies where cardiovascular events reported. ^5^For patients with time to mortality data only (excludes Clark 2002).

## Table S8 - Study level baseline demographic and health characteristics

| **Study** | **Total participants** | **Male; n/N (%)** | **Age (years)^1^; mean (SD); median [min, 25^th^ centile, 75^th^ centile, max]** | **Current smoker; n/N (%)** | **BMI^2^; mean (SD), n; median [IQR]** | **Total cholesterol (mmol/l); mean (SD), n** | **HDL cholesterol (mmol/l); mean (SD), n** |
| --- | --- | --- | --- | --- | --- | --- | --- |
| **AAA** | 3350 | 954/3350 (28) | 61.9 (6.6); 62 [50, 56, 67, 77] | 1085/3350 (32) | NR | 6.2 (1.1), 3330 | NR |
| **ARTPER** | 3748 | 1735/3748 (46) | 64.3 (8.9); 63 [49, 57, 71, 97] | 650/3748 (17) | 29.1 (4.7), 3743; 28.6 [25.9; 31.6] | 5.6 (1.0), 3722 | 1.4 (0.4), 3748 |
| **Kinmen Health Survey** | 1329 | 609/1329 (46) | 56.6 (11.1); 54 [40, 47, 65, 90] | 150/1329 (11) | 24.4 (3.5), 1329; 24.1 [22.0, 26.5] | 5.3 (1.0), 1309 | 1.3 (0.4), 1309 |
| **Mid Devon Pilot study** | 83 | 38/83 (46) | 64.9 (15.5); 69 [19, 57, 75, 86] | 13/83 (16) | 26.0 (4.1), 81; 25.7 [23.7, 28.0] | 6.1 (1.3), 23 | NR |
| **Mid Devon Hypertension cohort** | 247 | 112/247 (45) | 68.4 (10.0); 68 [47, 61, 77, 92] | 35/247 (14) | 28.0 (4.8), 213; 27.0 [25.0, 31.0] | 5.8 (1.2), 233 | 1.3 (0.5), 24 |
| **ViWoCo** | 1440 | 0/1440 (0) | 66.5 (5.2); 66 [59, 61, 71, 77] | 228/1440 (16) | 26.2 (5.3), 1410; 25.3 [22.8, 28.7] | 5.8 (1.0), 14440 | NR |
| **DARE** | 991 | 534/991 (54) | 63.0 (13.1), 65 [18, 56, 72, 91] | 106/983 (11) | 29.2 (5.2), 981; 28.0 [25.0, 32.0] | 5.3 (1.3), 425 | 1.6 (0.6), 308 |
| **EPIDEMCA** | 1029 | 403/1029 (39) | 73.8 (6.8); 72 [65, 68, 78, 99] | 131/1016 (13) | 21.5 (5.1), 956; 20.0 [18, 24] | 4.1 (1.0), 830 | NR |
| **Heinz Nixdorf** | 4814 | 2395/4814 (50) | 59.6 (7.8); 60 [45, 53, 66, 76] | 1128/4804 (23) | 28.0 (4.6), 4785; 27.0 [25.0, 30.0] | 5.9 (1.0), 4792 | 1.5 (0.4), 4789 |
| **InCHIANTI** | 1453 | 642/1453 (44) | 68.9 (15.7); 71 [21, 66, 78, 102] | 268/1185 (1361) | 27.2 (4.1), 1256; 27.0 [24.0, 30.0] | 5.6 (1.0), 1326 | 1.4 (0.4), 1326 |
| **Fuencarral Health Center** | 1361 | 520/1361 (38) | 69.6 (5.3); 70 [60, 66, 74, 79] | 145/1361 (11) | 29.1 (4.4), 1361; 29.0 [26.0, 32.0] | 5.5 (1.0), 1280 | 1.5 (0.4), 1280 |
| **LIFE** | 1635 | 537/1635 (33) | 78.4 (5.2); 78 [70, 74, 83, 90] | 52/1628 (3) | 30.2 (6.0), 1635; 29.4 [26.0, 33.5] | 4.6 (1.0), 1532 | 1.6 (0.5), 1532 |
| **Limburg** | 3035 | 1464/(48) | 58.2 (9.4); 58 [40, 51, 65, 78] | 1467/3022 (49) | 26.7 (4.3), 3018; 26.0 [24.0, 29.0] | NR | NR |
| **Look AHEAD** | 342 | 138/342 (40) | 56.1 (7.1); 56 [45, 51, 60, 75] | 16/341 (5) | 36.4 (5.9), 342; 36 [32, 40] | 5.1 (0.9), 342 | 1.1 (0.3), 342 |
| **LRC** | 624 | 279/624 (45) | 66.0 (10.3); 69 [38, 59, 74, 81] | 373/624 (60) | 24.8 (3.7), 623; 24.0 [22.0, 27.0] | 5.6 (1.0), 623 | 1.5 (0.4), 623 |
| **MARK** | 2495 | 1529/2495 (61) | 62.1 (7.7); 62 [34, 56, 67, 74] | 714/2495 (29) | 29.3 (4.6), 2495; 28.7 [26.2, 31.7] | 5.8 (1.1), 2495 | 1.3 (0.3), 2492 |
| **MESA** | 6814 | 3213/6814 (47) | 62.2 (10.2); 62 [44, 53, 70, 84] | 887/6792 (13) | 28.3 (5.5), 6814; 28 [15, 25, 31, 62] | 5.0 (0.9), 6791 | 1.3 (0.4), 6788 |
| **Men born in 1914** | 480 | 480/480 (100) | 68 (0); 68 [68, 68, 68, 68] | 170/479 (35) | 25.1 (3.3), 477; 24.7 [15.6, 23.1, 27.2, 38.8] | 6.0 (1.1), 477 | NR |
| **San Diego** | 2404 | 824/2404 (34) | 58.8 (11.4); 59 [29, 49, 69, 91] | 144/2404 (6) | 27.0 (5.3), 2397; 26.1 [16.2, 23.4, 29.6, 56.4] | 5.4 (1.1), 2327 | 1.4 (0.4), 2327 |
| **Elderly Chinese** | 3121 | 1380/3121 (44) | 68.4 (7.4); 67 [58, 62, 74, 93] | 789/3121 (25) | 23.7 (3.6), 3121; 23 [12, 21, 26, 42] | 5.7 (1.4), 3121 | NR |
| **SMART** | 11,139 | 7375/11,139 (66) | 56.7 (12.4); 58 [18, 49, 66, 82] | 3216/11,055 (29) | 26.9 (4.4), 11,121’ 26.3 [14.3, 24.0, 29.1, 58.8] | 5.1 (1.4), 11,080 | 1.3 (0.4), 11,062 |
| **SUMMIT** | 596 | 403/596 (68) | 66.9 (9.1); 67 [41, 61, 73, 88] | 42/596 (7) | 30.2 (5.7), 594; 29.4 [18.4, 26.3, 33.4, 55.5] | 4.4 (1.1), 357 | 1.4 (0.4), 340 |
| **VIETNAM** | 4462 | 4462/4462 (100) | 37.8 (2.5); 38 [31, 36, 39, 48] | 1934/4458 (43) | 26.9 (4.5), 4461; 26.3 [15.7, 23.9, 29.1, 66.8] | 5.5 (1.1), 4462 | 1.2 (0.3), 4462 |
| **WALCS** | 442 | 206/442 (47) | 70.0 (8.1); 69 [55, 64, 76, 93] | 34/408 (8) | 29.0 (6.1), 439; 28.3 [17.0, 24.7, 32.1, 59.6] | 4.6 (0.9), 406 | 1.2 (0.5), 406 |

NR: Not reported. **^1^**Number of participants with age available is as total number in study except for EPIDEMCA, where N=1024. ^2^Body Mass Index (kg/m^2^).

## Table S9 - Distribution of morbidities at baseline

| **Study** | **Total particip-ants** | **Hyperten-sion; n/N (%)** | **Diabetes; n/N (%)** | **Ischemic heart disease; n/N (%)** | **Cerebrovasc-ular disease; n/N (%)** | **Peripheral arterial disease; n/N (%)** | **Any cardiovasc-ular disease^1^; n/N (%)** | **Renal disease; n/N (%)** | **Atrial fibrillation/flutter; n/N (%)** |
| --- | --- | --- | --- | --- | --- | --- | --- | --- | --- |
| **AAA** | 3350 | 842/3350 (25) | 88/3262 (3) | 0/3350 (0) | 0/3350 (0) | 0/3350 (0) | 0/3350 (0) | 0/3350 (0) | 3/3350 (0) |
| **ARTPER** | 3748 | 1789/3748 (48) | 625/3748 (17) | 281/3748 (8) | 140/3748 (4) | 89/3659 (2) | 459/3748 (12) | NR | NR |
| **Kinmen Health Survey** | 1329 | 211/1275 (17) | 62/1213 (5) | NR | NR | NR | NR | NR | NR |
| **Mid Devon Pilot study** | 83 | 53/83 (64) | 50/83 (60) | 5/83 (6) | NR | NR | 5/83 (6) | NR | NR |
| **Mid Devon Hypertension cohort** | 247 | 247/247 (100) | 18/247 (7) | 44/247 (18) | 18/247 (7) | 5/247 (2) | 62/247 (25) | NR | 6/247 (2) |
| **ViWoCo** | 1440 | 675/1439 (47) | 113/1438 (8) | 99/1439 (7) | 84/1439 (6) | 2/1440 (0) | 168/1439 (12) | NR | 53/1440 (4) |
| **DARE** | 991 | 602/990 (61) | 709/991 (72) | 121/990 (12) | 63/990 (6) | 59/708 (8) | 200/990 (20) | 53/990 (5) | 26/991 (3) |
| **EPIDEMCA** | 1029 | 694/1016 (68) | 114/1004 (11) | NR | 27/1022 (3) | 45/1022 (4) | 67/1022 (7) | NR | NR |
| **Heinz Nixdorf** | 4814 | 2334/4796 (49) | 397/4814 (8) | 327/4799 (7) | 135/4785 (3) | 108/4732 (2) | 498/4799 (10) | NR | 80/4705 (2) |
| **InCHIANTI** | 1453 | 1074/1380 (78) | 187/1352 (14) | 81/1317 (6) | 80/1317 (6) | 151/1337 (11) | 262/1321 (20) | 19/265 (7) | 34/1286 (3) |
| **Fuencarral Health Center** | 1361 | 687/1361 (50) | 215/1361 (16) | 79/1361 (6) | 39/1361 (3) | 0/1361 (0) | 118/1361 (9) | 279/779 (36) | NR |
| **LIFE** | 1635 | 1360/1633 (83) | 424/1629 (26) | 490/1635 (30) | 185/1631 (11) | NR | 539/1635 (33) | NR | 38/1634 (2) |
| **Limburg** | 3035 | 1170/3024 (39) | 319/3030 (11) | 689/3033 (23) | 78/3027 (3) | NR | 736/3033 (24) | NR | NR |
| **Look AHEAD** | 342 | 279/342 (82) | 342/342 (100) | 36/342 (11) | 10/341 (3) | 3/342 (1) | 46/342 (13) | 17/342 (5) | NR |
| **LRC** | 624 | 44/624 (7) | 23/624 (4) | 58/624 (9) | 10/624 (2) | 10/624 (2) | 74/624 (12) | NR | NR |
| **MARK** | 2495 | 2122/2495 (85) | 808/2494 (32) | 0/2495 (0) | 0/2495 (0) | 0/2495 (0) | 0/2495 (0) | 138/2472 (6) | 42/2495 (2) |
| **MESA** | 6814 | 3508 /6814 (51) | 938/5853 (14) | 0/6814 (0) | 0/6814 (0) | 0/6814 (0) | 0/6814 (0) | 146/6789 (2) | 1/6765 (0) |
| **Men born in 1914** | 480 | 303/480 (63) | 30/476 (6) | 85/479 (18) | 27/480 (6) | NR | 107/480 (22) | NR | 19/480 (4) |
| **San Diego** | 2404 | 831/2401 (35) | 159/2401 (7) | 157/2404 (7) | 62/2404 (3) | NR | 203/2404 (8) | NR | NR |
| **Elderly Chinese** | 3121 | 1870/3121 (60) | 287/3121 (9) | 48/3121 (2) | 47/3121 (2) | NR | 48/3121 (2) | NR | NR |
| **SMART** | 11,139 | 8420/11,117 (76) | 2136/11,139 (19) | 752610,423 (72) | 2233/11,139 (20) | 1403/11,139 (13) | 7528/10,425 (72) | NR | NR |
| **SUMMIT** | 596 | 429/596 (72) | 473/596 (79) | 172/596 (29) | 75/596 (13) | 40/596 (7) | 237/596 (30) | 7/596 (1) | 22/595 (4) |
| **VIETNAM** | 4462 | 2377/4462 (53) | 237/4462 (5) | 65/4462 (1) | 5/4462 (0) | 65/4462 (1) | 65/4462 (1) | 0/4462 (0) | NR |
| **WALCS** | 442 | 315/442 (71) | 98/442 (22) | 147/442 (33) | 26/442 (6) | 0/442 (0) | 157/442 (36) | NR | NR |

NR: Not reported. ^1^At least one of ischemic heart disease, cerebrovascular disease or peripheral arterial disease, or a generic diagnosis of cardiovascular disease.

## Table S10 Modified QUIPS judgements for included studies

| **Study name** | **Reviewer** | **Study Participation** | **Study Attrition** | **Blood pressure measurement** | **Outcome Measurement** | **Blinded Agreement** | **Discussion Agreement** | **Overall rating after discussion** |
| --- | --- | --- | --- | --- | --- | --- | --- | --- |
| **Mid Devon** | A | Low | Unclear | Low | Low | N | Y | Unclear |
|  | B | Unclear | Unclear | High | High |  |  |  |
|  |  |  |  |  |  |  |  |  |
| **Mid Devon HT** | A | Low | Low | Low | Low | Y | Y | Low |
|  | B | Low | Low | Low | Low |  |  |  |
|  |  |  |  |  |  |  |  |  |
| **Elderly Chinese** | A | Low | Low | Low | Low | Y | Y | Low |
|  | C | Low | Low | Low | Low |  |  |  |
|  |  |  |  |  |  |  |  |  |
| **VIETNAM** | A | Low | Low | Low | Low | Y | Y | Low |
|  | C | Low | Low | Low | Low |  |  |  |
|  |  |  |  |  |  |  |  |  |
| **DARE** | A | Low | Low | Low | Low | N | Y | Low |
|  | B | Low | Unclear | Low | Unclear |  |  |  |
|  |  |  |  |  |  |  |  |  |
| **AAA** | A | Low | Low | Low | Low | Y | Y | Low |
|  | B | Low | Low | Low | Low |  |  |  |
|  |  |  |  |  |  |  |  |  |
| **InCHIANTI** | A | Low | Low | Unclear | Unclear | N | Y | Unclear |
|  | B | Low | Unclear | Low | Low |  |  |  |
|  |  |  |  |  |  |  |  |  |
| **Heinz Nixdorf** | A | Low | Unclear | Low | Low | N | Y | Low |
|  | C | Low | Low | Low | Low |  |  |  |
|  |  |  |  |  |  |  |  |  |
| **SMART** | A | Low | Low | Low | Low | Y | Y | Low |
|  | C | Low | Low | Low | Low |  |  |  |
|  |  |  |  |  |  |  |  |  |
| **LRC** | A | Low | Unclear | Unclear | Low | N | Y | Low |
|  | C | Low | Unclear | Low | Low |  |  |  |
|  |  |  |  |  |  |  |  |  |
| **San Diego** | A | Low | Unclear | Low | Low | Y | Y | Low |
|  | C | Low | Unclear | Low | Low |  |  |  |
|  |  |  |  |  |  |  |  |  |
| **Fuencarral** | A | Low | Low | Low | Low | Y | Y | Low |
|  | C | Low | Low | Low | Low |  |  |  |
|  |  |  |  |  |  |  |  |  |
| **ARTPER** | A | Low | Low | Unclear | Low | N | Y | Low |
|  | C | Low | Low | Low | Low |  |  |  |
|  |  |  |  |  |  |  |  |  |
| **EPIDEMCA** | A | Unclear | Low | Unclear | Low | N | Y | Unclear |
|  | C | Low | Unclear | Low | Low |  |  |  |
|  |  |  |  |  |  |  |  |  |
| **MESA** | A | Low | Low | Low | Low | Y | Y | Low |
|  | C | Low | Low | Low | Low |  |  |  |
|  |  |  |  |  |  |  |  |  |
| **LIFE** | A | Unclear | Low | Low | Low | N | Y | Unclear |
|  | C | Low | Unclear | Low | Unclear |  |  |  |
|  |  |  |  |  |  |  |  |  |
| **Limburg PAOD** | A | Low | Unclear | Unclear | Low | N | Y | Low |
|  | C | Low | Low | Low | Low |  |  |  |
|  |  |  |  |  |  |  |  |  |
| **Men Born 1914** | A | Low | Low | Low | Low | Y | Y | Low |
|  | C | Low | Low | Low | Low |  |  |  |
|  |  |  |  |  |  |  |  |  |
| **Look AHEAD** | A | Low | Low | Low | Low | N | Y | Low |
|  | C | Unclear | Unclear | Low | Unclear |  |  |  |
| **Kinmen** | A | Low | Unclear | Unclear | Low | N | Y | Low |
|  | C | Low | Low | Low | Low |  |  |  |
|  |  |  |  |  |  |  |  |  |
| **WALCS** | A | Low | Unclear | Low | Low | N | Y | Low |
|  | C | Low | Unclear | Low | Unclear |  |  |  |
|  |  |  |  |  |  |  |  |  |
| **ViWoCo** | A | Low | Unclear | Low | Low | N | Y | Low |
|  | C | Low | Low | Low | Low |  |  |  |
|  |  |  |  |  |  |  |  |  |
| **SUMMIT** | C | Low | Low | Low | Unclear | N | Y | Low |
|  |  | *no relevant publication, reviewer C provided author judgments, 2nd review not feasible* | | | |  |  |  |
|  |  |  |  |  |  |  |  |  |
| **MARK** | A | Low | Unclear | Unclear | Unclear | N | Y | Unclear |
|  | C | Low | Unclear | Low | Unclear |  |  |  |
|  |  |  |  |  |  |  |  |  |
| **Summary** | **Low** | **43** | **28** | **39** | **38** |  |  | **19** |
|  | **Unclear** | **4** | **19** | **7** | **8** |  |  | **5** |
|  | **High** | **0** | **0** | **1** | **1** |  |  | **0** |

## Table S11 - Results of one-stage meta-analysis of observed plus imputed participant baseline data for all-cause mortality: hazard ratios for model including all selected covariates

|  | **Dataset including data from 23 studies, with observed and imputed baseline covariate data** | |
| --- | --- | --- |
| **Covariate** | **HR (95% CI)** | **p-value** |
| Systolic BP inter-arm difference (IAD) | 1.001 (1.000; 1.001) | 0.251 |
| Baseline systolic BP | 1.003 (1.002; 1.003) | <0.001 |
| Age | 1.101 (1.100; 1.102) | <0.001 |
| Sex (reference: female) | 1.674 (1.646; 1.704) | <0.001 |
| Smoking status | 1.605 (1.579; 1.631) | <0.001 |
| Body mass index (BMI) | 0.990 (0.988; 0.992) | <0.001 |
| Ethnic group^1^  (reference: white) | African American: 1.068 (0.981; 1.162)  Hispanic American: 0.896 (0.790; 1.016)  Other: 0.874 (0.776; 0.985) | Global: 0.031 |
| Total cholesterol | 0.980 (0.973; 0.986) | <0.001 |
| High density lipoprotein (HDL) | 0.866 (0.849; 0.884) | <0.001 |
| Hypertension diagnosis | 1.139 (1.119; 1.159) | <0.001 |
| Diabetes diagnosis | 1.652 (1.620; 1.684) | <0.001 |

^1^Black African and Chinese categories omitted from model.

## Table S12 - Results of one-stage meta-analysis of observed plus imputed data for cardiovascular mortality: hazard ratios for model including all selected covariates

|  | **Dataset including data from 23 studies, with observed and imputed baseline covariate data** | |
| --- | --- | --- |
| **Covariate** | **HR (95% CI)** | **p-value** |
| Systolic blood pressure inter-arm difference | 1.008 (1.006; 1.010) | <0.001 |
| Baseline SBP | 1.003 (1.003; 1.004) | <0.001 |
| Age | 1.116 (1.114; 1.118) | <0.001 |
| Sex (reference: female) | 1.670 (1.620; 1.722) | <0.001 |
| Smoking status | 1.861 (1.812; 1.911) | <0.001 |
| Body mass index (BMI) | 0.984 (0.981; 0.988) | <0.001 |
| Ethnic group^1^  (reference: white) | African American: 1.621 (1.376; 1.909)  Hispanic American: 1.139 (0.875; 1.484)  Other: 1.505 (1.244; 1.820) | Global: <0.001 |
| High density lipoprotein (HDL) | 0.674 (0.650; 0.699) | <0.001 |
| Hypertension diagnosis | 1.451 (1.404; 1.499) | <0.001 |
| Diabetes diagnosis | 1.594 (1.544; 1.646) | <0.001 |

^1^Black African and Chinese categories omitted from model.

## Table S13 - Results of one-stage meta-analysis of observed plus imputed data for cardiovascular fatal and non-fatal events: hazard ratios for model including all selected covariates

|  | **Dataset including data from 23 studies, with observed and imputed baseline covariate data** | |
| --- | --- | --- |
| **Covariate** | **HR (95% CI)** | **p-value** |
| Systolic blood pressure inter-arm difference | 1.002 (1.001; 1.003) | <0.001 |
| Baseline SBP | 1.005 (1.004; 1.005) | <0.001 |
| Age | 1.053 (1.052; 1.054) | <0.001 |
| Sex (reference: female) | 1.691 (1.662; 1.720) | <0.001 |
| Smoking status | 1.370 (1.349; 1.392) | <0.001 |
| Body mass index (BMI) | 0.995 (0.994; 0.997) | <0.001 |
| Ethnic group^1^  (reference: white) | African-American: 0.869 (0.788; 0.959)  Hispanic- American: 0.847 (0.743; 0.966)  Other: 0.717 (0.622; 0.826) | Global: <0.001 |
| Total cholesterol | 1.009 (1.002; 1.015) | <0.001 |
| High density lipoprotein (HDL) | 0.654 (0.641; 0.668) | <0.001 |
| Hypertension diagnosis | 1.403 (1.379; 1.428) | <0.001 |
| Diabetes diagnosis | 1.344 (1.318; 1.371) | <0.001 |

^1^Black African and Chinese categories omitted from model.


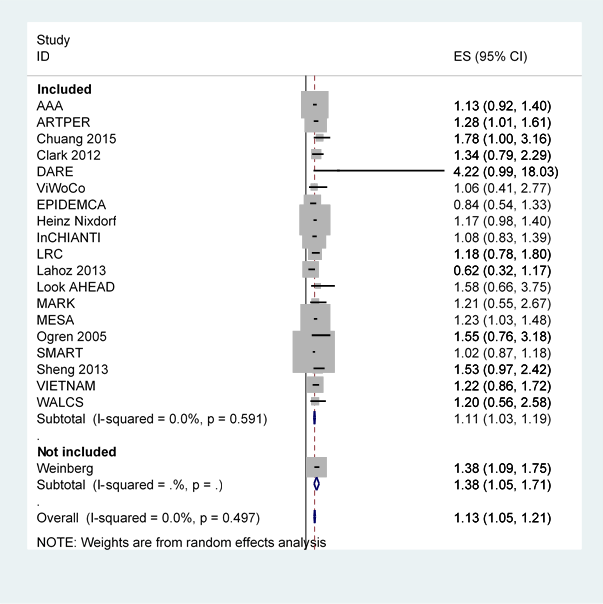


Hazard ratio of systolic inter-arm difference ≥10mmHg for cardiovascular events adjusted for age, sex, smoking status, diabetes hypertension and total cholesterol, for comparison with published hazard ratio for Weinberg et al. Shaded areas represent study weights.

## Figure S1 – Forest plot of cardiovascular mortality hazard ratios for included and non-included studies

Effect size = ln(Hazard Ratio) from study-specific Cox regression analyses. Egger’s test *p =* 0.657

0

0.01

0.02

0.03

0.04

Standard error of effect size

-0.1

-0.05

0

0.05

0.1

Effect size

## Figure S2 - Funnel plot derived from the two-stage all-cause mortality model in all 23 studies including continuous systolic inter-arm difference as the only covariate

Studies in parentheses were not included in analyses due to missing outcome data

*for risk score analyses, numbers restricted to those falling within eligible age ranges and free from cardiovascular disease at baseline

## Figure S3 - Contributions of cohorts to individual analyses


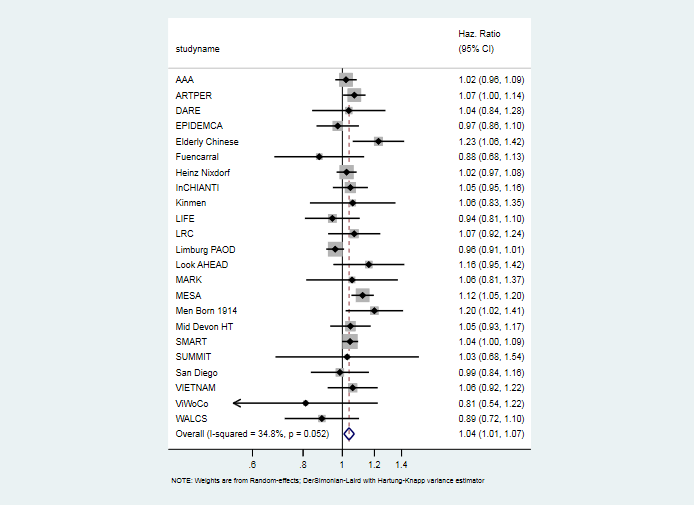


Overall P-value = 0.014

## Figure S4 - Forest plot of continuous systolic inter-arm difference, per 5mmHg increment, with adjustment for baseline systolic BP, age and sex: time to all-cause mortality

NOTE: Weights are from random effects analysis

Overall (I-squared = 78.3%, p = 0.000)

ViWoCo

ID

DARE

MARK

SMART

Kinmen

Heinz Nixdorf

ARTPER

EPIDEMCA

Look AHEAD

Fuencarral

Elderly Chinese

VIETNAM

LIFE

WALCS

Men Born 1914

MESA

SUMMIT

Mid Devon HT

LRC

Study

InCHIANTI

AAA

San Diego

0.97 (0.88, 1.06)

0.39 (-0.15, 0.94)

ES (95% CI)

0.94 (0.26, 1.61)

1.28 (0.72, 1.84)

0.76 (0.66, 0.85)

0.86 (0.71, 1.01)

1.09 (0.98, 1.20)

0.99 (0.86, 1.11)

0.82 (0.57, 1.07)

0.77 (0.30, 1.24)

0.97 (0.55, 1.38)

1.36 (1.19, 1.53)

0.92 (0.55, 1.29)

0.81 (0.52, 1.10)

1.18 (0.76, 1.59)

0.75 (0.32, 1.18)

0.95 (0.88, 1.03)

0.66 (-0.18, 1.49)

0.89 (0.61, 1.17)

0.84 (0.64, 1.04)

1.35 (1.22, 1.48)

0.89 (0.75, 1.03)

1.12 (0.97, 1.27)

100.00

2.06

Weight

1.48

1.97

7.03

6.34

6.85

6.67

4.87

2.53

2.96

6.03

3.35

4.32

2.96

2.86

7.25

1.04

4.44

5.57

%

6.60

6.47

6.36

0.97 (0.88, 1.06)

0.39 (-0.15, 0.94)

ES (95% CI)

0.94 (0.26, 1.61)

1.28 (0.72, 1.84)

0.76 (0.66, 0.85)

0.86 (0.71, 1.01)

1.09 (0.98, 1.20)

0.99 (0.86, 1.11)

0.82 (0.57, 1.07)

0.77 (0.30, 1.24)

0.97 (0.55, 1.38)

1.36 (1.19, 1.53)

0.92 (0.55, 1.29)

0.81 (0.52, 1.10)

1.18 (0.76, 1.59)

0.75 (0.32, 1.18)

0.95 (0.88, 1.03)

0.66 (-0.18, 1.49)

0.89 (0.61, 1.17)

0.84 (0.64, 1.04)

1.35 (1.22, 1.48)

0.89 (0.75, 1.03)

1.12 (0.97, 1.27)

100.00

2.06

Weight

1.48

1.97

7.03

6.34

6.85

6.67

4.87

2.53

2.96

6.03

3.35

4.32

2.96

2.86

7.25

1.04

4.44

5.57

%

6.60

6.47

6.36

0

-1.84

0

1.84

Internal–external cross validation analysis using set of variables in final model, excluding one study out of 23 in turn to run model and derive calibration slope for model including prognostic indicator in excluded study, indicating acceptable performance of the model using all data.

## Figure S5 - Random effects meta-analysis of calibration slope for all-cause mortality.


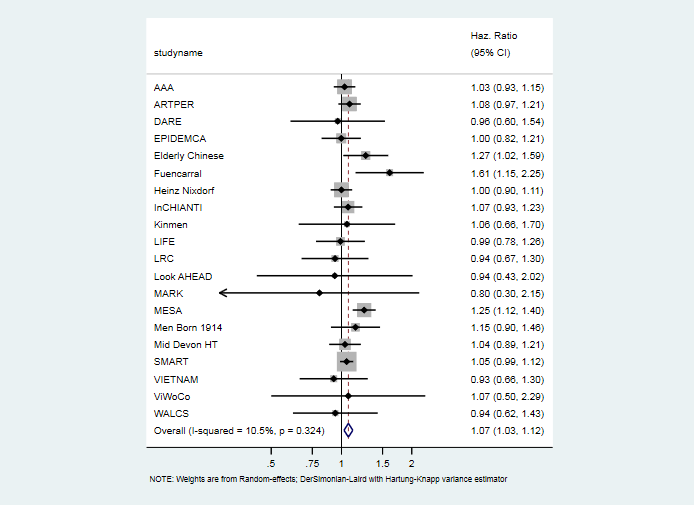


Overall P-value = 0.004

## Figure S6 - Forest plot of continuous systolic inter-arm difference, per 5mmHg increment, with adjustment for baseline systolic BP, age and sex: time to cardiovascular mortality

NOTE: Weights are from random effects analysis

Overall (I-squared = 74.8%, p = 0.000)

Look AHEAD

Mid Devon HT

EPIDEMCA

LRC

ViWoCo

Elderly Chinese

MARK

Fuencarral

InCHIANTI

Heinz Nixdorf

SMART

Kinmen

MESA

SUMMIT

DARE

Men Born 1914

ARTPER

VIETNAM

ID

WALCS

LIFE

AAA

Study

0.97 (0.83, 1.10)

0.93 (0.10, 1.76)

1.04 (0.70, 1.38)

0.58 (0.28, 0.88)

0.95 (0.57, 1.33)

1.03 (-0.14, 2.20)

1.56 (1.32, 1.79)

0.59 (-0.70, 1.88)

1.02 (-0.11, 2.14)

1.22 (1.07, 1.37)

1.15 (0.97, 1.32)

0.67 (0.55, 0.78)

0.84 (0.57, 1.11)

0.90 (0.76, 1.03)

-0.25 (-1.76, 1.25)

0.87 (0.40, 1.34)

1.26 (0.74, 1.77)

1.05 (0.83, 1.26)

0.92 (0.24, 1.59)

ES (95% CI)

0.88 (0.28, 1.48)

0.89 (0.47, 1.31)

0.84 (0.61, 1.06)

100.00

1.99

5.52

6.08

5.10

1.13

6.84

0.95

1.21

7.80

7.54

8.13

6.38

7.99

0.72

4.23

3.77

7.09

2.69

Weight

3.16

4.69

6.99

%

0.97 (0.83, 1.10)

0.93 (0.10, 1.76)

1.04 (0.70, 1.38)

0.58 (0.28, 0.88)

0.95 (0.57, 1.33)

1.03 (-0.14, 2.20)

1.56 (1.32, 1.79)

0.59 (-0.70, 1.88)

1.02 (-0.11, 2.14)

1.22 (1.07, 1.37)

1.15 (0.97, 1.32)

0.67 (0.55, 0.78)

0.84 (0.57, 1.11)

0.90 (0.76, 1.03)

-0.25 (-1.76, 1.25)

0.87 (0.40, 1.34)

1.26 (0.74, 1.77)

1.05 (0.83, 1.26)

0.92 (0.24, 1.59)

ES (95% CI)

0.88 (0.28, 1.48)

0.89 (0.47, 1.31)

0.84 (0.61, 1.06)

100.00

1.99

5.52

6.08

5.10

1.13

6.84

0.95

1.21

7.80

7.54

8.13

6.38

7.99

0.72

4.23

3.77

7.09

2.69

Weight

3.16

4.69

6.99

%

0

-2.2

0

2.2

Internal–external cross validation analysis using set of variables in final model, excluding one study out of 21 in turn to run model and derive calibration slope for model including prognostic indicator in excluded study, indicating acceptable performance of the model using all data.

## Figure S7 - Random effects meta-analysis of calibration slope for cardiovascular mortality


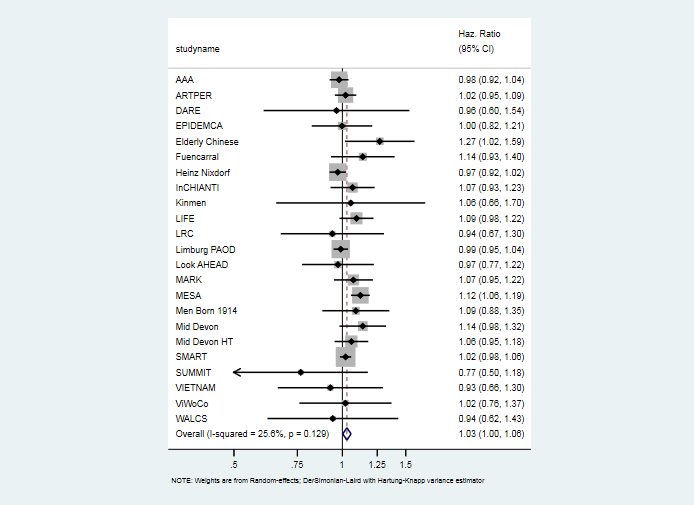


Overall P-value = 0.033

## Figure S8 - Forest plot of cardiovascular fatal or non-fatal first events within 10 years for continuous systolic inter-arm difference, per 5mmHg increment, with adjustment for baseline systolic BP, age and sex

*Lower risk scores truncated since thresholds unchanged on adjustment for inter-arm difference*

## Figure S9 - Modified ten-year risk score tables showing adjusted risk scores according to systolic inter-arm difference
