# Supplementary figures and images for: Associations Between Systolic Interarm Differences in Blood Pressure and Cardiovascular Disease Outcomes and Mortality: Individual Participant Data Meta-Analysis, Development and Validation of a Prognostic Algorithm: The INTERPRESS-IPD Collaboration
Source: Hypertension. 2020 Dec 21;77(2):650–61. doi: 10.1161/HYPERTENSIONAHA.120.15997 (PMC7803446; doi:10.1161/HYPERTENSIONAHA.120.15997)

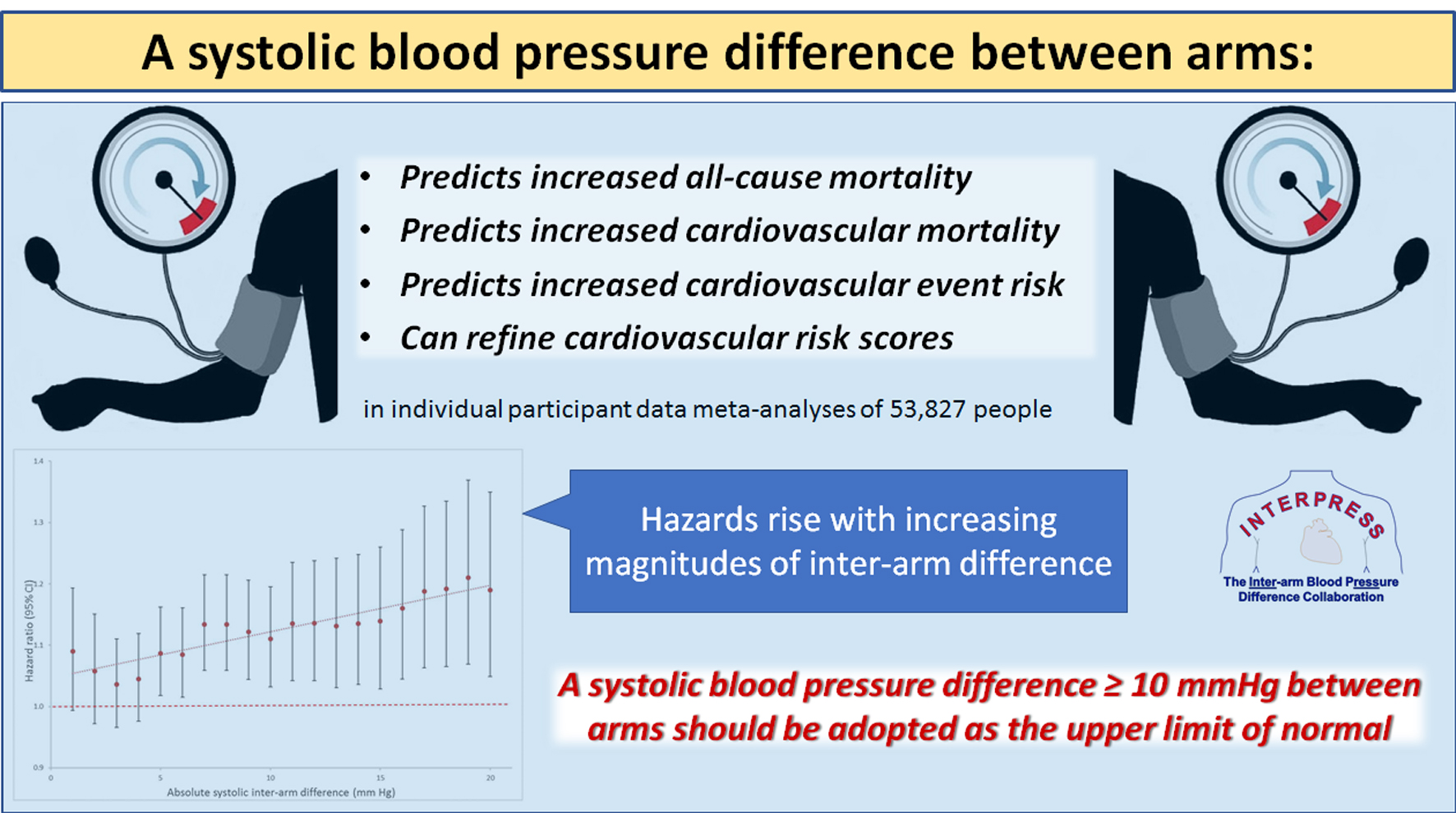

Supplement: Supplementary file 2 [file hyp-77-650-s002.jpg]
