# Supplementary material for: Associations Between Systolic Interarm Differences in Blood Pressure and Cardiovascular Disease Outcomes and Mortality: Individual Participant Data Meta-Analysis, Development and Validation of a Prognostic Algorithm: The INTERPRESS-IPD Collaboration
Source: Hypertension. 2020 Dec 21;77(2):650–61. doi: 10.1161/HYPERTENSIONAHA.120.15997 (PMC7803446; doi:10.1161/HYPERTENSIONAHA.120.15997)
Supplement: Supplementary file 4 [file hyp-77-650-s004.pdf]

**From:**	Clark, Chris
**To:**	Hypertension
**Subject:**	RE: HYPE/2020/15997R1: Request Final Files -style issues
**Date:**	Tuesday, November 10, 2020 4:43:55 AM
**Attachments:**	Final Hypertension TIFF.zip
**Importance:**	High

*** **CAUTION:** This email originated from outside of the **American Heart Association**. Do **not** click links or open attachments unless you recognize the sender and know the content is safe. ***

Dear Renata

I hope that I have addressed your comments, **please see replies below.** I will attend to any final issues by return. The upload link supplied only provides for updated text files so I attach revised figures to this email. Please let me know if I need to do anything else.

BW

Chris

Chris Clark
I am working from home at present. My usual academic days are Tuesday to Thursday
Tel +44 (0) 771 414 6710
or leave me a message on my office number: +44 (0)1392 722754

From: hypertension@heart.org <hypertension@heart.org>
Sent: 09 November 2020 20:23
To: Clark, Chris <C.E.Clark@exeter.ac.uk>
Subject: HYPE/2020/15997R1: Request Final Files -style issues

CAUTION: This email originated from outside of the organisation. Do not click links or open attachments unless you recognise the sender and know the content is safe.

PLEASE READ: During this unprecedented and challenging time, the health and safety of you, your family, and your community is of utmost importance. We appreciate that one aspect of this current situation is the inability to continue research work and that some measures will lead to a significant hindering of research progress. Please know that we are flexible regarding turnaround times for revisions and other tasks during this stressful time and deadlines will be extended as needed. If you are able to, please contact us if you need any extensions or if you experience any challenges around manuscript preparation; we will work with you.

09-Nov-2020

Dr. Christopher E. Clark
University of Exeter Medical School

Primary Care Research Group
Room 111, Smeall Building, St Luke's Campus
Magdalen Road
Exeter, Devon EX1 2LU
UNITED KINGDOM

MS ID#: HYPE/2020/15997R1
MS TITLE: Associations between systolic inter-arm differences in blood pressure and cardiovascular disease outcomes and mortality

Dear Dr. Clark:

Thanks for addressing the style issues. However there are some items missing:

- Figures - the resolution is low (=95 and 72 dpi). Provide them as TIFF or EPS files .Figures should be supplied at the highest resolution possible. Line Art: 1200dpi. Everything else (CT scans, radiographs, other halftones and color figures): at least 600dpi.
Please see attached zip file – all TIFF files are now 1200 dpi

- Figure 2 - rotate clockwise the Y-axis numbers.
Done – see fig 2

- Legend of tables S2 and S5 refers to several references in the text of the manuscript. Please provide permission to reprint or include a statement that permission is not necessary (and why) by email.
The only citations for tables S2 and S5 are the primary data sources – all are listed in the supplemental reference list and all appear in the reference list of the main text.
I do not fully understand what permission you think should be provided. The data sources all originated with one or more collaborating authors, and as stated all have signed data sharing agreements. Please explain if I have misunderstood.

- Rename Funding statement by Sources of Funding.
Done

- Rename the Competing interests statement by Disclosures.
Done

- Text of the manuscript has to have only one set of references located after Disclosures. Remove the 26 references from the title page and place them in the reference section.
These are not references – they indicate the authors' institutions

- Online supplement - same as above. It has to have one complete set of references numbered from 1 to......located before supplemental tables.
These are not references – they indicate the authors' institutions

- Single space the text of the online supplement.
Done

In addition, all figures must be publication ready prior to final submission. Please refer to https://www.ahajournals.org/pb-assets/migration/Hype_InstructionsforFigureSubmission-1532530896710.pdf for figure requirements.

Authorship changes are not allowed post acceptance. If you make any changes in authorship of your manuscript (additions, deletions, change of order) prior to acceptance, you must complete a Change of Authorship Form and have all authors sign agreeing to the change. If your manuscript is accepted, it will not be possible to publish it until all signatures are in place. The form may be downloaded from https://www.ahajournals.org/pb-assets/migration/COA_Form-1532532203410.pdf.

The files are needed before your manuscript can enter the production cycle. When all listed items are complete please submit online at https://hype-submit.aha-journals.org/cgi-bin/main.plex?el=A7Hm3CnRT4B3HOq6BS5A9ftdxjhNXsX2MhSwG3cwN692nwZ.

Please contact our editorial office if you have questions about the items requested (hypertension@heart.org). We will not be able to submit your manuscript for publication until final materials are received in this editorial office. Also, the date your final materials are received will be published in Hypertension as the acceptance date for your manuscript.

Thanks again for submitting your interesting work to Hypertension. We look forward to receiving future manuscripts from you. With kind regards,

Sincerely,

Renata Gil
Assistant Managing Editor
Hypertension Editorial Office

==
To submit, go to https://hype-submit.aha-journals.org/cgi-bin/main.plex?el=A7Hm3CnRT4B3HOq6BS5A9ftdxjhNXsX2MhSwG3cwN692nwZ

//

Dear Renata

I have now uploaded the final revised files for this paper. Please let me know if they are acceptable.

I would like to clarify one point from the feedback below: "- Legend of tables 1 and 2 refers to reference 1 in the text of the manuscript. Please provide permission to reprint or include a statement that permission is not necessary (and why) by email."
I can clarify that these are not citations but footnotes - I have substituted asterisks for clarity.

BW

Chris

Chris Clark
